# Quantifying the Number of Pregnancies at Risk of Malaria in 2007: A Demographic Study

**DOI:** 10.1371/journal.pmed.1000221

**Published:** 2010-01-26

**Authors:** Stephanie Dellicour, Andrew J. Tatem, Carlos A. Guerra, Robert W. Snow, Feiko O. ter Kuile

**Affiliations:** 1 Child and Reproductive Health Group, Liverpool School of Tropical Medicine, Liverpool, United Kingdom; 2 Malaria Public Health and Epidemiology Group, Centre for Geographic Medicine, Kenyan Medical Research Institute–University of Oxford–Wellcome Trust Collaborative Programme, Nairobi, Kenya; 3 Department of Geography and Emerging Pathogens Institute, University of Florida, Gainesville, Florida, United States of America; 4 Spatial Ecology and Epidemiology Group, Department of Zoology, University of Oxford, Oxford, United Kingdom; 5 Centre for Tropical Medicine, Nuffield Department of Clinical Medicine, University of Oxford, CCVTM, Oxford, United Kingdom; 6 Department of Infectious Diseases, Tropical Medicine & AIDS, Academic Medical Centre, University of Amsterdam, Amsterdam, The Netherlands; University of Queensland Centre for Clinical Research, Australia

## Abstract

**Background:**

Comprehensive and contemporary estimates of the number of pregnancies at risk of malaria are not currently available, particularly for endemic areas outside of Africa. We derived global estimates of the number of women who became pregnant in 2007 in areas with *Plasmodium falciparum* and *P. vivax* transmission.

**Methods and Findings:**

A recently published map of the global limits of *P. falciparum* transmission and an updated map of the limits of *P. vivax* transmission were combined with gridded population data and growth rates to estimate total populations at risk of malaria in 2007. Country-specific demographic data from the United Nations on age, sex, and total fertility rates were used to estimate the number of women of child-bearing age and the annual rate of live births. Subregional estimates of the number of induced abortions and country-specific stillbirths rates were obtained from recently published reviews. The number of miscarriages was estimated from the number of live births and corrected for induced abortion rates. The number of clinically recognised pregnancies at risk was then calculated as the sum of the number of live births, induced abortions, spontaneous miscarriages, and stillbirths among the population at risk in 2007. In 2007, 125.2 million pregnancies occurred in areas with *P. falciparum* and/or *P. vivax* transmission resulting in 82.6 million live births. This included 77.4, 30.3, 13.1, and 4.3 million pregnancies in the countries falling under the World Health Organization (WHO) regional offices for South-East-Asia (SEARO) and the Western-Pacific (WPRO) combined, Africa (AFRO), Europe and the Eastern Mediterranean (EURO/EMRO), and the Americas (AMRO), respectively. Of 85.3 million pregnancies in areas with *P. falciparum* transmission, 54.7 million occurred in areas with stable transmission and 30.6 million in areas with unstable transmission (clinical incidence <1 per 10,000 population/year); 92.9 million occurred in areas with *P. vivax* transmission, 53.0 million of which occurred in areas in which *P. falciparum* and *P. vivax* co-exist and 39.9 million in temperate regions with *P. vivax* transmission only.

**Conclusions:**

In 2007, 54.7 million pregnancies occurred in areas with stable *P. falciparum* malaria and a further 70.5 million in areas with exceptionally low malaria transmission or with *P. vivax* only. These represent the first contemporary estimates of the global distribution of the number of pregnancies at risk of *P. falciparum* and *P. vivax* malaria and provide a first step towards a more informed estimate of the geographical distribution of infection rates and the corresponding disease burden of malaria in pregnancy.

*Please see later in the article for the Editors' Summary*

## Abstract 2

By combining data from the Malaria Atlas Project with country-specific data, Feiko ter Kuile and colleagues provide the first contemporary global estimates of the annual number of pregnancies at risk of malaria.

## Editors' Summary

**Background:**

Malaria, a mosquito-borne parasitic disease, is a major global public-health problem. About half of the world's population is at risk of malaria, which kills about one million people every year. Most of these deaths are caused by *Plasmodium falciparum*, which thrives in tropical and subtropical regions. However, the most widely distributed type of malaria is *P. vivax* malaria, which also occurs in temperate regions. Most malaria deaths are among young children in sub-Saharan Africa, but pregnant women and their unborn babies are also very vulnerable to malaria. About 10,000 women and 200,000 babies die annually because of malaria in pregnancy, which can cause miscarriages, preterm births, and low-birth-weight births. Over the past decade, a three-pronged approach has been developed to prevent and control malaria in pregnancy. This approach consists of intermittent preventative treatment of pregnant women with antimalarial drugs, the use of insecticide-treated bed nets to protect pregnant women from the bites of infected mosquitoes, and management of malarial illness among pregnant women.

**Why Was This Study Done?:**

This strategy has begun to reduce the burden of malaria among pregnant women and their babies but the resources available for its introduction are very limited in many of the developing countries where malaria is endemic (always present). Policy makers in these countries need to know the number of pregnancies at risk of malaria so that they can use their resources wisely. However, although the World Health Organization recently estimated that more than 30 million African women living in malaria endemic areas become pregnant and are at risk for malaria each year, there are no comprehensive and contemporary estimates of the number of pregnancies at risk of malaria for endemic areas outside Africa. In this study, the researchers derive global estimates of the number of women who became pregnant in 2007 in areas with *P. falciparum* and *P. vivax* transmission.

**What Did the Researchers Do and Find?:**

The researchers estimated the sizes of populations at risk of malaria in 2007 by combining maps of the global limits of *P. vivax* and *P. falciparum* transmission with data on population densities. They used data from various sources to calculate the annual number of pregnancies (the sum of live births, induced abortions, miscarriages, and still births) in each country. Finally, they calculated the annual number of pregnancies at risk of malaria in each country by multiplying the number of pregnancies in the entire country by the fraction of the population living within the spatial limits of malaria transmission in that country. In 2007, they calculate, 125.2 million pregnancies occurred in areas with *P. falciparum* and/or *P. vivax* transmission. These pregnancies—60% of all pregnancies globally—resulted in 82.6 million live births. 77.4 million at-risk pregnancies occurred in Southeast Asia and the Western Pacific (India had the most pregnancies at risk of both *P. falciparum* and *P. vivax* malaria), 30.3 million in Africa, 13.1 million in Europe and the Eastern Mediterranean, and 4.3 million in the Americas. 54.7 million at-risk pregnancies occurred in regions with stable *P. falciparum* transmission (more than one case of malaria per 10,000 people per year), whereas 70.5 million occurred in areas with low malaria transmission or *P. vivax* transmission only.

**What Do These Findings Mean?:**

These findings are the first contemporary estimates of the global distribution of the number of pregnancies at risk of *P. falciparum* and *P. vivax* malaria. They do not provide any information on the actual incidence of malaria during pregnancy or the health burden on mothers and unborn babies. They simply represent “any risk” of exposure. So, for example, the researchers calculate that only about 5,000 actual malaria infections may occur annually among the 70.5 million at-risk pregnancies in areas with very low malaria transmission or with *P. vivax* transmission only. Furthermore, these findings do not allow for the seasonality of malaria—pregnancies that occur outside of the transmission season may be at no or very low risk of malaria. Nevertheless, the estimates reported in this study are an important first step towards a spatial map of the burden of malaria in pregnancy and should help policy makers allocate resources for research into and control of this important public-health problem.

**Additional Information:**

Please access these Web sites via the online version of this summary at http://dx.doi.org/10.1371/journal.pmed.1000221.

- Information is available from the World Health Organization on malaria and on malaria in pregnancy (in several languages)
- The US Centers for Disease Control and Prevention also provides information on malaria and on malaria in pregnancy (in English and Spanish)
- Information is available from the Roll Back Malaria Partnership on all aspects of global malaria control, including information on malaria in pregnancy
- The Malaria in Pregnancy Consortium is undertaking research into the prevention and treatment of malaria in pregnancy and also provides a comprehensive bibliographic database of published and unpublished literature relating to malaria in pregnancy
- The Malaria Atlas Project provides maps of malaria transmission around the world
- MedlinePlus provides links to additional information on malaria (in English and Spanish)

## Introduction

Malaria in pregnancy can have devastating consequences to a pregnant woman and the developing fetus, but comprehensive estimates of the annual number of women who become pregnant each year in malaria endemic areas and are therefore at risk of malaria are not available, particularly for Latin America and the Asia-Pacific regions. These figures are an important first step towards informing policy makers and for estimating the regional needs for therapeutic and disease prevention tools for malaria in pregnancy. The most cited global estimate is from the Roll Back Malaria Partnership, which states that “each year approximately 50 million women living in malaria endemic countries throughout the world become pregnant” [1]. However, an explanation of the methods used to derive these estimates is not provided. More comprehensive estimates exist for Africa and are provided by the Africa Regional Office (AFRO) of the World Health Organization (WHO) in their widely quoted strategic framework document for malaria prevention and control during pregnancy in the African region [2]. Their estimate of 24.6 million pregnancies at risk of malaria (predominantly *P. falciparum*), is based on the number of live born babies delivered in malarious areas of Africa in the year 2000 using a combination of malaria risk maps [3] and estimates of the number of live births from UNICEF [4]. A more recent estimate by the WHO states that “In Africa, 30 million women living in malaria endemic areas become pregnant each year” [5]. Estimates for outside of Africa are less clear, particularly for *P. vivax*. *P. vivax* is the most widely distributed human malaria parasite and co-occurs with *P. falciparum* in tropical areas but also occurs in temperate regions outside the limits of *P. falciparum* transmission. It is the major cause of malaria in much of Asia and Latin America [6],[7], and recent evidence has shown that *P. vivax* infections are far from benign and can result in significant morbidity in pregnant women with serious consequences for maternal and infant health [8]–[10].

Here we define a global estimate of the number of pregnancies at risk of *P. falciparum* and *P. vivax* malaria in 2007 by combining malaria spatial limits developed by the Malaria Atlas Project (MAP; www.map.ox.ac.uk), which define the total population at risk of malaria [11], with country-specific demographic data on women of childbearing age provided by the United Nations and published data on induced abortions and spontaneous pregnancy loss.

## Methods

### Data Sources

#### The global limits of *P. falciparum* malaria

The initial focus of the Malaria Atlas Project has been *P. falciparum* [12] due to its global epidemiological significance [13] and better prospects for its control and local elimination [14]. The global spatial limits of *P. falciparum* malaria transmission in 2007 have recently been mapped. This was done by triangulating data on transmission exclusion using biological rules based on temperature and aridity limits on the bionomics of locally dominant *Anopheles* vectors, data on nationally reported case incidence rates, and other medical intelligence [15]. The resulting map stratifies the malaria endemic world by stable and unstable transmission in 2007 [15]. Unstable transmission refers to areas where transmission is plausible biologically, but limited, with a clinical incidence of less than one case per 10,000 population per year. Stable transmission refers to areas with a minimum of one clinical case per 10,000 population per year [15].

#### The global limits of *P. vivax* malaria

Initial attempts to map the limits of *P. falciparum* and *P. vivax* transmission were made by Guerra et al. [16],[17]. The resulting maps and “masks” (mapped areas that are filtered and excluded from analyses) used were later tested against the Malaria Atlas Project parasite prevalence database to assess their feasibility [18],[19]. This testing revealed that the accuracy to define areas of zero transmission risk due to very low population densities was limited because of the coarse spatial resolution of the initial map. Moreover, in the initial mapping [16],[17], a high density population mask was used on the basis of the assumption that no transmission occurs in areas where the population density is so high that conditions become unsuitable for transmission through the process of urbanization. However, recent analyses [19] provide evidence suggesting that high density population masks and urban extent maps should not be used to map zero risk because some transmission can occur in high density urban areas, although this is significantly lower than in rural areas [18],[19]. Therefore, for the current analyses, the *P. vivax* limits were redefined using the same methods as in Guerra et al. [16],[17], but without applying the population-based masks. Also, previously excluded *P. vivax* endemic countries have now been added after a more extensive review of the literature; these include Comoros, Djibouti, Madagascar, and Uzbekistan. This refinement of the spatial limits of transmission for *P. vivax* accounted for an approximate 19% increase in the population at risk (PAR) compared with previous estimates [16],[17], principally (18%) due to the inclusion of major cities.

#### Gridded population data

The Global Rural-Urban Mapping Project (GRUMP) alpha version provides gridded population counts and population density estimates for the years 1990, 1995, and 2000, both adjusted and unadjusted to the United Nations' national population estimates [11],[20]. The adjusted population counts for the year 2000 were projected to 2007 by applying national, medium variant, intercensal growth rates by country [21] using methods described previously [22].

#### Annual number of pregnancies per country

The number of pregnancies was calculated as the sum of the number of live births, induced abortions, and spontaneous pregnancy loss (including miscarriages and stillbirths) in 2007.

#### Live births

The annual number of live births in 2007 was estimated per country using demographic data on the proportion of women of childbearing age (WOCBAs) within a population and the total fertility rates. The data were abstracted from the United Nations' national population estimates, which provide publicly accessible demographic information by year, age, sex, and country for Africa, Asia, and the Americas [23]. The number of WOCBAs in each country, defined as the mid-year resident number of women aged between 15 and 49y, was obtained for the years 2005 and 2010 (interim years are not available), and the number of WOCBAs for 2007 was calculated as the midpoint between the 2005 and 2010 estimates. The fraction of WOCBAs per country was then calculated as the number of WOCBAs in 2007 divided by the mid-year resident population at risk in 2007 (available by year).

The total fertility rate (TFR) is an age-standardised measure of fertility and corresponds to the total number of children that would be born alive to a woman entering her childbearing years at age 15y if she lived to the end of her childbearing years (age 49y) and if her fertility during these 35 reproductive years was the same as the average woman of childbearing age. The total fertility rate divided by 35 is the average number of live births per WOCBA per year and when multiplied by 1,000 this is expressed as the rate of live births per 1,000 WOCBAs per year.

#### Induced abortions, miscarriages, and stillbirth rates

Subregional data on induced abortion rates were obtained from a recently published review that calculated the worldwide, regional, and subregional incidence of safe and unsafe abortions in women of child bearing age in 2003 by use of reports from official national reporting systems, nationally representative demographic health surveys, hospital data, other surveys, and published studies [24].

Country-specific information on stillbirth rates was abstracted from model-based estimates published by Stanton et al. [25] that derived data from vital registration, demographic and health surveys (DHS), and data from study reports integrated into a regression model. Regional estimates were used for three malaria endemic countries for which country-specific estimates were not available (French Guiana, Mayotte, and Timor-Leste).

Country-specific data on miscarriages (spontaneous abortions) are not available. To calculate the proportion of pregnancies resulting in miscarriage, a method was applied that uses multipliers to work backwards from the (known) number of live births and induced abortions to recover the (unknown) underlying number of pregnancies that “produced” them, as described in detail previously [24],[26]–[28]. The method takes account of pregnancies that are terminated voluntarily during the period of risk for miscarriage and estimates the number of spontaneous pregnancy loss (stillbirths and miscarriages) as 10% of induced abortions plus 20% of live births. It is based on clinical studies of rates of pregnancy loss by gestational age that indicate that for each 100 induced abortions an additional ten clinically recognised pregnancies will have aborted spontaneously prior to the average gestational age of induced abortions in that population, and that approximately 120 additional clinically recognised pregnancies are required to “produce” 100 live births [27],[28]. For example, in Afghanistan it was estimated that in 2007 1.182 million live births occurred among a population of 27 million and a further 0.284 million induced abortions occurred. The number of spontaneous pregnancy losses (the sum of the number of stillbirths and miscarriages) was therefore estimated at 0.2×1,182 plus 0.1×0.284 = 0.265 million, and the total number of pregnancies as 1.731 million. The reported number of miscarriages used in this manuscript represents the number of spontaneous pregnancy losses calculated through the multiplier method as described above, minus the country-specific number of stillbirths obtained from the review by Stanton et al. [25]. The estimates provided in this study refer to clinically recognised pregnancies and do not take into account the potentially large but unknown rates of embryonic loss that may occur in the first 4–6 wk of gestation.

#### Estimating the annual number of pregnancies exposed to malaria

To obtain the total population at risk, the limits of stable and unstable *P. falciparum* transmission and the limits of *P. vivax* transmission described above were overlaid onto the Global Rural-Urban Mapping Project (GRUMP) alpha surface, projected to 2007. For every malaria endemic country of the world, the population within each set of limits was extracted, following approaches described previously [13]. The number of pregnancies at risk of malaria was then calculated from the total annual number of pregnancies estimated to have occurred in 2007 in the entire country multiplied by the fraction of the total resident population living within the spatial limits of malaria transmission in that country.

## Results

Tables 1 and 2 provide a summary of the total population living within the global spatial limits of malaria transmission in 2007, and the corresponding number of total population, pregnancies, and live births, stratified by species and transmission patterns (within areas of assumed unstable and stable *P. falciparum* transmission), globally and by WHO region.

**Table 1:** Demographic data for malaria endemic countries.

WHORO Region	*n* of MECs	Total Population (Both Sexes)^a^	WOCBAs^a^	Total *n* of Pregnancies^b^	TPR^c^	Pregnancy Rate per 1,000 WOCBAs§	Percentage Pregnancies Ending in:			
							Live-births	Still-births	Spontaneous Abortions	Induced Abortions
**AFRO**	43	755	178	36	7.16	204	72.4%	2.3%	13.3%	11.9%
**EMRO/EURO**	19	544	142	19	4.76	136	68.8%	2.3%	13.1%	15.8%
**AMRO**	21	530	143	16	3.81	109	63.2%	0.9%	14.0%	22.0%
**SEARO/WPRO**	19	3,327	881	91	3.62	103	62.4%	1.6%	13.1%	22.8%
**Global**	102	5,157	1,343	162	4.23	121	65.5%	1.8%	13.3%	19.5%

a Source: United Nations Development Program (in millions).

b The total number of pregnancies is the sum of the number of live-births, stillbirths, spontaneous, and induced abortions (in millions).

c The total pregnancy rate (TPR) and the annual pregnancy rate per 1,000 WOCBAs are weighted means per region and is for illustration purposes only. The number of pregnancies was derived directly as the sum of the national estimates within each region and globally.

MEC, malaria endemic countries; WHORO, World Health Organization Regional Office.

**Table 2:** Total population at risk of *P. falciparum* and/or *P. vivax* malaria by WHO regional office in 2007 (in millions) (percent of the population in malaria endemic countries at risk).

WHORO Region	*P. falciparum* Transmission^a^			*P. vivax* Transmission^a^	Any Species
	Stable Transmission^b^	Unstable Transmission^b^	Overall	Overall	Overall
**AFRO**	599.9 (79.4)	8.4 (1.1)	607.8 (80.5)	73.2 (9.7)	615.4 (81.5)
**EMRO/EURO**	89.8 (16.5)	101.7 (18.7)	190.9 (35.1)	285.1 (52.4)	343.9 (63.2)
**AMRO**	41.2 (7.8)	50.2 (9.5)	91.4 (17.2)	96.2 (18.2)	138.2 (26.1)
**SEARO/WPRO**	654.9 (19.7)	824.9 (24.8)	1479.3 (44.5)	2,722.3 (81.8)	2,770.1 (83.3)
**Global**	1,385.8 (26.9)	985.1 (19.1)	2,369.4 (45.9)	3,176.9 (61.6)	3,867.6 (75.0)

Similar tables with risk estimates by continent and by pregnancy outcome (live-birth, induced abortions, stillbirths, and miscarriages) are provided in Tables S1, S2, S3.

a Includes countries where *P. falciparum* and *P. vivax* co-exist.

b Stable transmission, ≥1 autochthonous *P. falciparum* cases per 10,000 people per annum; unstable transmission, <1 autochthonous *P. falciparum* cases per 10,000 people per annum [15].

MEC, malaria endemic countries; TPR, total pregnancy rate; WHORO, World Health Organization Regional Office.

**Figure 1:**
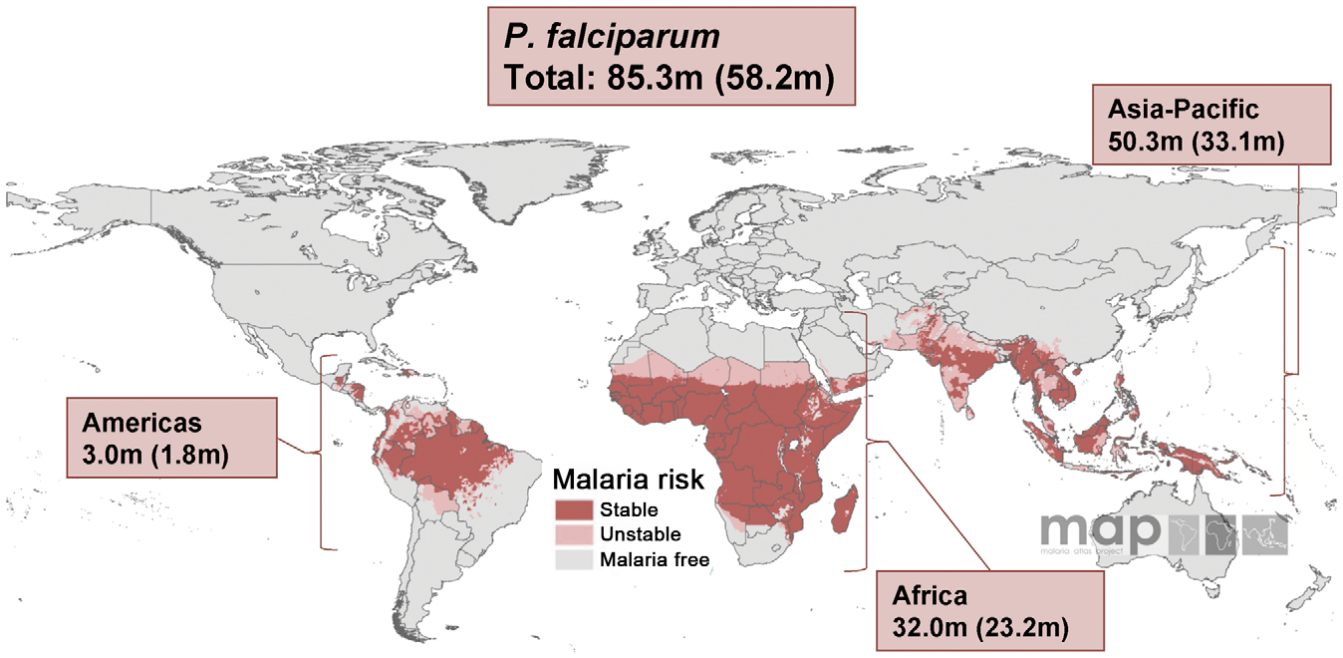
Malaria risk map for *P. falciparum* and corresponding number of pregnancies in each continent in 2007.

**Figure 2:**
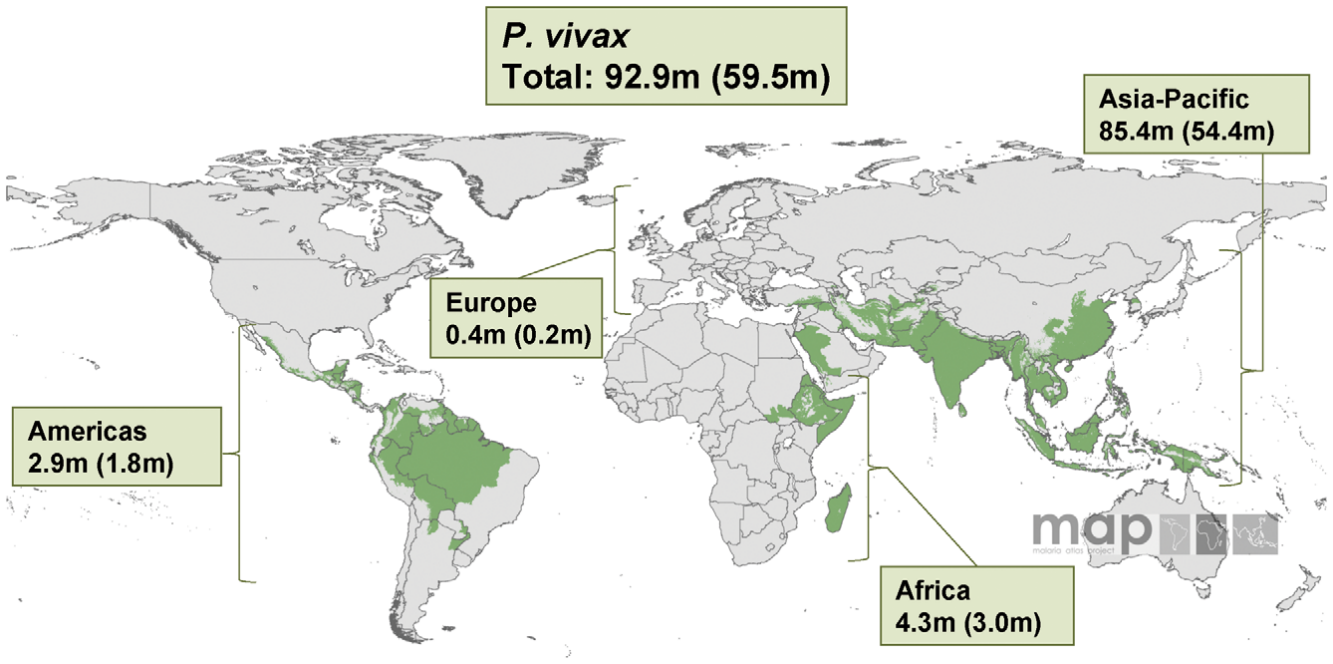
Malaria risk map for *P. vivax* and corresponding number of pregnancies in each continent in 2007.

The compiled data showed that, globally, 125.2 million women living in areas with *P. falciparum* and/or *P. vivax* transmission became pregnant in 2007: 77.4 million (61.8%) in the countries that fall under the regional office of the WHO for the South East Asian (SEARO) and the Western Pacific Region (WPRO); 30.3 million (24.2%) in AFRO; 13.1 million (10.5%) in the Eastern Mediterranean and European Region (EMRO and EURO); and only 4.3 million (3.4%) in the American Region (AMRO) (Table 3). Figures 1 and 2 display the same analysis by species, but depicted by continent rather than by WHO region. Of the 125.2 million pregnancies, 82.6 million (66.0%) are estimated to result in live births; 48.8 million (63.0%), 22.1 million (72.7%), 9.0 million (68.8%), and 2.7 million (63.1%) in the SEARO/WPRO, AFRO, EMRO/EURO, and AMRO regions, respectively (Table 4). It illustrates that the proportional distribution of pregnancies at risk resulting in live births is slightly different from the distribution of total pregnancies at risk, primarily reflecting the differences in the proportion of pregnancies ending in induced abortions, which is much lower in the AFRO region (11.9%) compared to the global average in the malaria endemic countries of 19.5% [24].

**Table 3:** Number of pregnancies at risk of *P. falciparum* and/or *P. vivax* malaria by WHO regional office in 2007 (in millions) (column %).

WHORO Region	*P. falciparum* Transmission^a^			*P. vivax* Transmission^a^	Any Species
	Stable Transmission^b^	Unstable Transmission^b^	Overall	Overall	Overall
**AFRO**	29.6 (54.1)	0.4 (1.2)	30.0 (35.1)	3.6 (3.9)	30.3 (24.2)
**EMRO/EURO**	4.0 (7.3)	4.2 (13.7)	8.2 (9.6)	10.4 (11.2)	13.1 (10.5)
**AMRO**	1.4 (2.5)	1.6 (5.2)	3.0 (3.5)	2.9 (3.1)	4.3 (3.4)
**SEARO/WPRO**	19.7 (36.1)	24.5 (79.9)	44.2 (51.8)	76.0 (81.8)	77.4 (61.8)
**Global**	54.7	30.6	85.3	92.9	125.2

Similar tables with risk estimates by continent and by pregnancy outcome (live-birth, induced abortions, stillbirths, and miscarriages) are provided in Tables S1, S2, S3.

a Includes countries where *P. falciparum* and *P. vivax* co-exist.

b Stable transmission, ≥1 autochthonous *P. falciparum* cases per 10,000 people per annum; unstable transmission, <1 autochthonous *P. falciparum* cases per 10,000 people per annum [15].

MEC, malaria endemic countries; TPR, total pregnancy rate; WHORO, World Health Organization Regional Office.

**Table 4:** Number of live-births born to pregnancies at risk of at risk of *P. falciparum* and/or *P. vivax* malaria by WHO regional office in 2007 (in millions) (column %).

WHORO Region	*P. falciparum* Transmission^a^			*P. vivax* Transmission^a^	Any Species
	Stable Transmission^b^	Unstable Transmission^b^	Overall	Overall	Overall
**AFRO**	21.6 (56.7)	0.3 (1.3)	21.8 (37.4)	2.5 (4.3)	22.1 (26.7)
**EMRO/EURO**	2.8 (7.3)	2.9 (14.1)	5.6 (9.7)	7.1 (12.0)	9.0 (10.9)
**AMRO**	0.8 (2.2)	1.0 (5.0)	1.8 (3.2)	1.8 (3.1)	2.7 (3.3)
**SEARO/WPRO**	12.9 (33.8)	16.1 (79.5)	28.9 (49.7)	48.0 (80.6)	48.8 (59.1)
**Global**	38.0	20.2	58.2	59.5	82.6

Similar tables with risk estimates by continent and by pregnancy outcome (live-birth, induced abortions, stillbirths, and miscarriages) are provided in Tables S1, S2, S3.

a Includes countries where *P. falciparum* and *P. vivax* co-exist.

b Stable transmission, ≥1 autochthonous *P. falciparum* cases per 10,000 people per annum; unstable transmission, <1 autochthonous *P. falciparum* cases per 10,000 people per annum [15].

MEC, malaria endemic countries; TPR, total pregnancy rate; WHORO, World Health Organization Regional Office.

### *P. falciparum* Malaria

**Figure 3:**
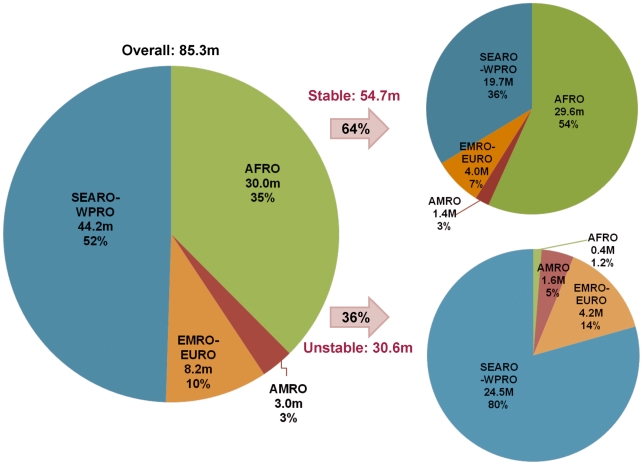
Distribution of the number of pregnancies in areas with *P. falciparum* malaria in 2007 by WHO regions and the corresponding proportion living under stable versus unstable transmission. Blue, SEARO and WPRO; green, AFRO; orange, EMRO; red, AMRO.

Of the 125.2 million pregnancies defined above, 85.3 million occur in areas with *P. falciparum* transmission, 51.8% of them (44.2 million) are in the combined SEARO-WPRO regions and 35.1% (30.0 million) in the AFRO region. The remainder live in the EMRO-EURO (9.6%) and AMRO regions (3.5%) (Figure 3; Table 3). As expected, the top five ranked countries with the highest number of pregnancies at risk of *P. falciparum* malaria were the malaria endemic countries with the largest overall populations: India (28.2 million), Nigeria (6.5 million), Indonesia (4.4 million), Pakistan (3.7 million), and the Democratic Republic of the Congo (3.3 million). Overall, 64.1% of 85.3 million pregnancies at risk of *P. falciparum* malaria live in areas with assumed stable transmission (Figure 3). However, this varies widely by region; from 98.7% in the AFRO region to none in the EURO region. As depicted in Figure 3, 55.3% of the 44.2 million pregnancies at risk of *P. falciparum* in the WPRO/SEARO region occur in areas of very low and unstable transmission.

### *P. vivax* Malaria

**Figure 4:**
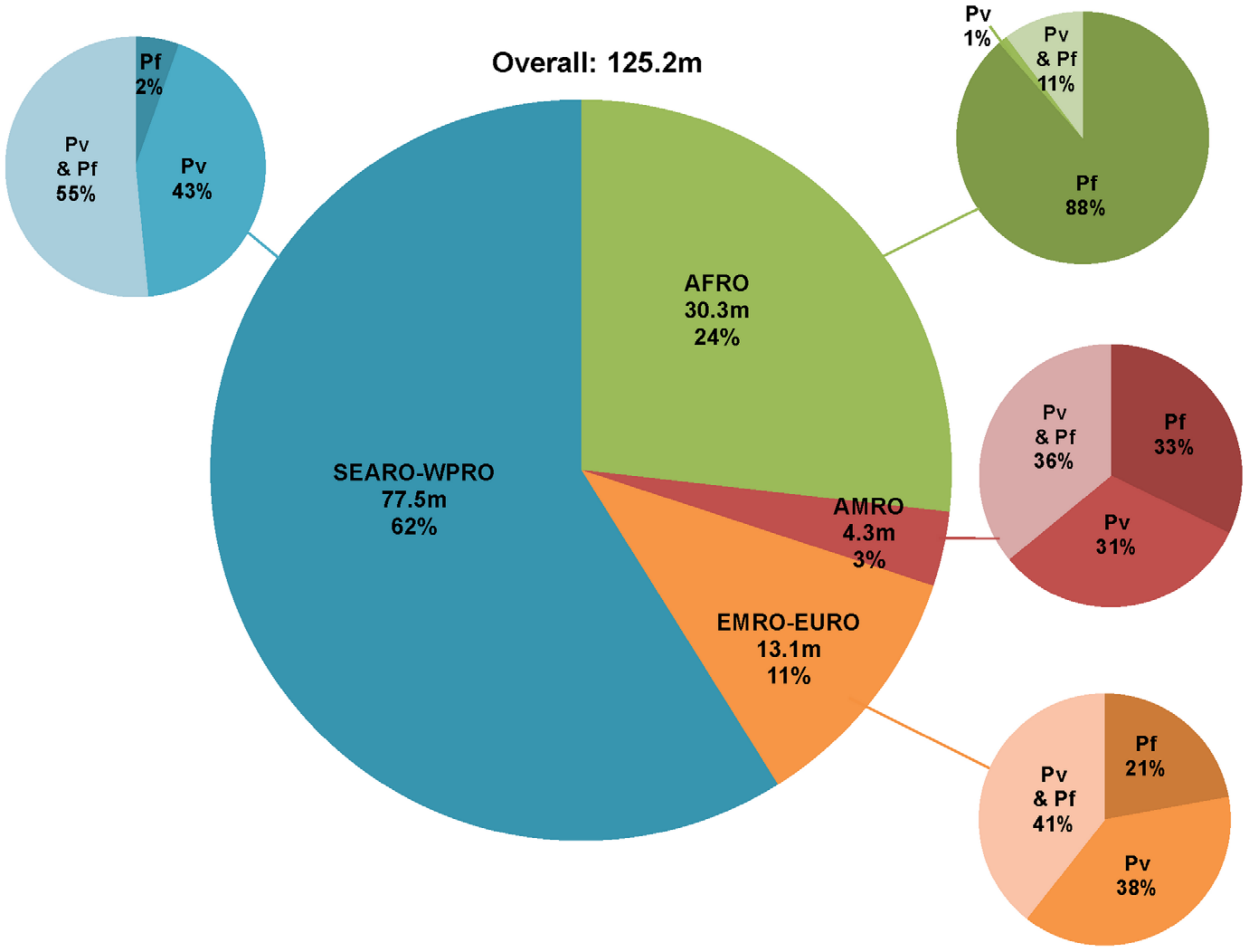
Distribution of the number of pregnancies in malaria endemic areas in 2007 by WHO regions and by species (*P. vivax* transmission only, *P. falciparum* transmission only or transmission of both species). Blue, SEARO and WPRO; green, AFRO; orange, EMRO; red, AMRO. *Pv*, *P. vivax*; *Pf*, *P. falciparum*.

Globally, an estimated 92.9 million pregnancies occurred in areas endemic for *P. vivax* in 2007 (including in areas where both *P. falciparum* and *P. vivax* co-exist) (Figure 2). The top five ranked countries include: India (32.9 million), China (21.2 million), Indonesia (6.3 million), Pakistan (5.8 million), and Bangladesh (4.7 million). In the WPRO/SEARO region, where the majority of the populations at risk of *P. vivax* live (Figure 4), approximately 98.2% of those pregnancies in malaria endemic countries occur in areas with *P. vivax* transmission (alone or combined with *P. falciparum*). By contrast this was only 11.9% for the AFRO region where *P. vivax* transmission is principally restricted to the horn of Africa region, Madagascar, and the Comoros islands (Figure 4).

The country-specific demographic data and population at risk estimates (Table S1), as well as total pregnancies at risk and by specific pregnancy outcomes (live births, induced abortions, stillbirths, and miscarriages; Table S2) and summary estimates by other regional categories (continents instead of WHO regions; Table S3), are provided as supplemental information. In addition, information is provided illustrating which countries are included in the different WHO regions (also see Figure S1) [29]. In brief, all malaria endemic countries on the African continent fall under the Africa Regional Office (AFRO), with the exception of Djibouti, Somalia, and Sudan, which fall under the EMRO office.

## Discussion

This is the first time, to our knowledge, that contemporary species-specific estimates of the annual number of pregnancies at risk of malaria globally have been made. Our findings suggest that in 2007 approximately 125 million pregnancies occurred in areas with *P. falciparum* and/or *P. vivax* transmission, resulting in 83 million live births; representing approximately 60% of all pregnancies globally. Approximately 85 million pregnancies occurred in areas with *P. falciparum* transmission and 93 million in areas with transmission of *P. vivax* transmission, of which about 53 million occurred in areas where both species co-exist. The pregnancies at risk estimates for *P. falciparum* and *P. vivax* in Africa (32 million [30 million in the WHO-AFRO region]) are consistent with the previous estimates by WHO (25–30 million). By contrast, the numbers at risk outside Africa are much higher (95 million) than previously estimated (25 million). Comparisons between the estimates produced in this study and the previous WHO estimates are made difficult because details of the methodology used by the WHO is not provided and it is not clear if they included all transmission areas or only areas with stable malaria transmission. Inclusion of only those areas with stable *P. falciparum* transmission in our study resulted in global risk estimates of just less than 55 million pregnancies, 31 million in Africa and 23 million in the other regions, i.e., very similar to the previous WHO estimates. However, the numbers of pregnancies at risk outside Africa increase almost 4-fold if areas with unstable *P. falciparum* transmission are included (clinical incidence <1 per 10,000 population/year) (30 million) and areas situated in the temperate regions outside the limits of *P. falciparum* transmission that have *P. vivax* transmission only (40 million) are also included. It is also not clear if the previous WHO estimates included pregnancies resulting in live births only or included adjustments for induced abortions or spontaneous pregnancy loss. Since only approximately two-thirds of all pregnancies result in live births, estimates that include all pregnancies are about one-third higher than estimates based on live births only.

Although risk estimates are widely quoted figures, it is important to place them in perspective. The estimates provided here merely define the global distribution of pregnancies that occur within the global spatial limits of malaria transmission. These estimates therefore represent “any risk” of exposure to malaria during pregnancy, and do not represent the distribution of actual incidence or health burden on mothers and unborn babies, which is beyond the scope of this paper. More than half (71 million) of the 125 million pregnancies occur in areas with unstable *P. falciparum* transmission (31 million) or with transmission of *P. vivax* only (40 million), and the risk of acquiring malaria in these areas is extremely low. Thus, although these 71 million pregnancies represent more than 50% of the global number of pregnancies at risk, they may only contribute a small proportion to the number of infections in pregnancy. For example, if the actual incidence of malaria infection in these very low transmission areas is 1 in 10,000 per person-year (52 wk), and if the average pregnancy resulting in a live birth takes 38 wk from fertilisation to term, then 71 million pregnancies at risk may result in only 5,188 actual malaria infections, whereas in areas with infection rates of 1.36 or higher per person-year, all term pregnancies have been potentially exposed to malaria. Furthermore, the definition of stable transmission for *P. falciparum* used included all areas with more than one clinical case per 10,000 population per year. This included almost all pregnancies at risk in the AFRO Region (99% of the 30 million pregnancies at risk) and 25 million of the 95 million (26%) pregnancies in the other WHO regions. However, these stable transmission strata cover a very wide range of transmission intensities and the actual risk of infection to the 55 million individuals and the impact on maternal and infant health varies enormously within this range.

At the higher end of the transmission spectrum, the majority of malaria infections in pregnancy remain asymptomatic or pauci-symptomatic, yet are a major cause of severe maternal anaemia and preventable low birth weight, especially in the first and second pregnancies. In areas with stable, but low transmission, and certainly in areas with unstable and exceptionally low transmission, infections can become severe in all gravidae groups because most women of childbearing age in these regions have low levels of pre-pregnancy and pregnancy-specific protective immunity to malaria [30]. The most recent version of the World Malaria Map [28] from the Malaria Atlas Project shows that 89% of the populations in stable *P. falciparum* areas outside Africa live in areas characterised by low malaria endemicity (defined as *P. falciparum* parasite rate in children 2–10 y of age of ≤5%). This total includes all of the stable *P. falciparum* transmission areas in the Americas, and 88% of the populations at risk in the Central and South-East Asia-Pacific region [31]. Our estimates do not take seasonality into account and include all pregnancies occurring throughout the year, whereas those pregnancies that occur outside of the transmission season may be at no risk, or very low risk of exposure.

Our risk estimates for *P. vivax* are likely to be less accurate than those for *P. falciparum* because of greater uncertainties about the basic biology of transmission and clinical epidemiology. For example, the climatic constraints on *P. vivax* transmission are less well defined, the accuracy of clinical reporting of *P. vivax* in areas with coincidental *P. falciparum* is poor, and the untreated hypnozoite stage of *P. vivax*, which can remain dormant in infected liver cells for months or years, provides an additional challenge to the interpretation of prevalence and incidence data [15]. We used a refined *P. vivax* risk map that resulted in a 19% increase over previous population at risk estimates (adjusted for population growth) [18],[19], principally resulting from the removal of the population density masks and thereby the inclusion of many large cities. In most of these cities, pregnancies will be at low or very low risk of autochthonous infections. Imported malaria associated with travel to rural areas may be a greater risk factor in these cities. We did not consider infections with *P. ovale* or *P. malariae*, as their distribution is not well described and the adverse effects on maternal health and the newborn infant are unknown.

In the current analysis we used the map of the global spatial limits of *P. falciparum* malaria, which stratifies the malaria endemic world by stable and unstable transmission published in 2008 [15].This map uses a simple divide between very low risk and higher transmission intensities and a crude proxy to account for the corresponding levels of acquired immunity in women of childbearing age. As a next step, we will examine the burden of malaria in pregnancy in terms of health impact on the pregnant women (e.g., febrile episodes, impact on maternal anaemia and maternal mortality), the newborn baby (e.g., impact on the frequency of preterm births and low birth-weight) and the infant (e.g., susceptibility to malaria). For this project, we will use the more refined *P. falciparum* transmission intensity model of risk within the defined stable limits which was developed recently by the Malaria Atlas Project [31], allowing disease impact calculations across multiple transmission strata to be made. It is also important to take the different pregnancy outcomes into account in these further burden estimates. Of the 125 million pregnancies, one in five are estimated to be terminated voluntarily during the period of risk for miscarriage, and only about two-thirds (82.6 millions) are expected to result in live births. Although malaria in pregnancy is associated with miscarriages and stillbirths [30], the majority of the health and economic burden is likely through the impact on pregnancies that result in live births by increasing the risk of preterm births and low birth-weight [30] and by modifying the susceptibility to malaria in the infant [32]–[34].

Most of the existing research and policy guidance for malaria control in pregnancy has focussed on *P. falciparum* in the stable transmission regions of sub-Saharan Africa. The results of this study are consistent with the previous WHO-RBM risk estimates for areas with stable *P. falciparum* malaria in Africa, but our work offers advancement on the existing risk estimates for malaria endemic countries outside Africa. In these regions, the burden of malaria in pregnancy is less well defined, both in terms of the number of pregnancies and its actual impact on health. Policy guidelines for malaria control in pregnancy are also less well developed for these regions.

These estimates of the number of pregnancies at risk of malaria provide a first step towards a spatial map of the burden of malaria in pregnancy and a more informed platform with which to estimate the associated disease and economic impact and its geographical distribution. Such global estimates provide guidance in terms of priority setting for resource allocation for both research and policy for the control of malaria in pregnancy. This project provides a dynamic framework that allows risk estimates to be updated when new risk maps of *P. falciparum* and *P. vivax* become available as the world attempts to move towards malaria elimination and eradication.

## Supporting Information

Figure S1 Map of the WHO Regions (http://www.who.int/about/regions/en/index.html).
(1.07 MB TIF)

Table S1 Demographic characteristics and total population at risk of *P. falciparum* and/or *P. vivax* malaria by malaria endemic country and by WHO regional office in 2007 (in millions).
(0.38 MB PDF)

Table S2 Total number of pregnancies by pregnancy outcome in areas with *P. falciparum* and/or *P. vivax* transmission by continent in 2007 (in millions).
(0.54 MB PDF)

Table S3 Total population, number of pregnancies, and number of live-births born to pregnancies in malaria endemic countries by continent in 2007 (in millions).
(0.28 MB PDF)

## Abbreviations

AFRO: Regional Office for Africa
AMRO: Regional Office for the Americas
EMRO: Regional Office for the Eastern Mediterranean
EURO: Regional Office for Europe
SEARO: Regional Office for South- East Asia
WHO: World Health Organization
WOCBA: women of childbearing age
WPRO: Regional Office for the Western Pacific
