# Supplementary figures and images for: Quantifying the Number of Pregnancies at Risk of Malaria in 2007: A Demographic Study
Source: PLoS Med. 2010 Jan 26;7(1):e1000221. doi: 10.1371/journal.pmed.1000221 (PMC2811150; doi:10.1371/journal.pmed.1000221)

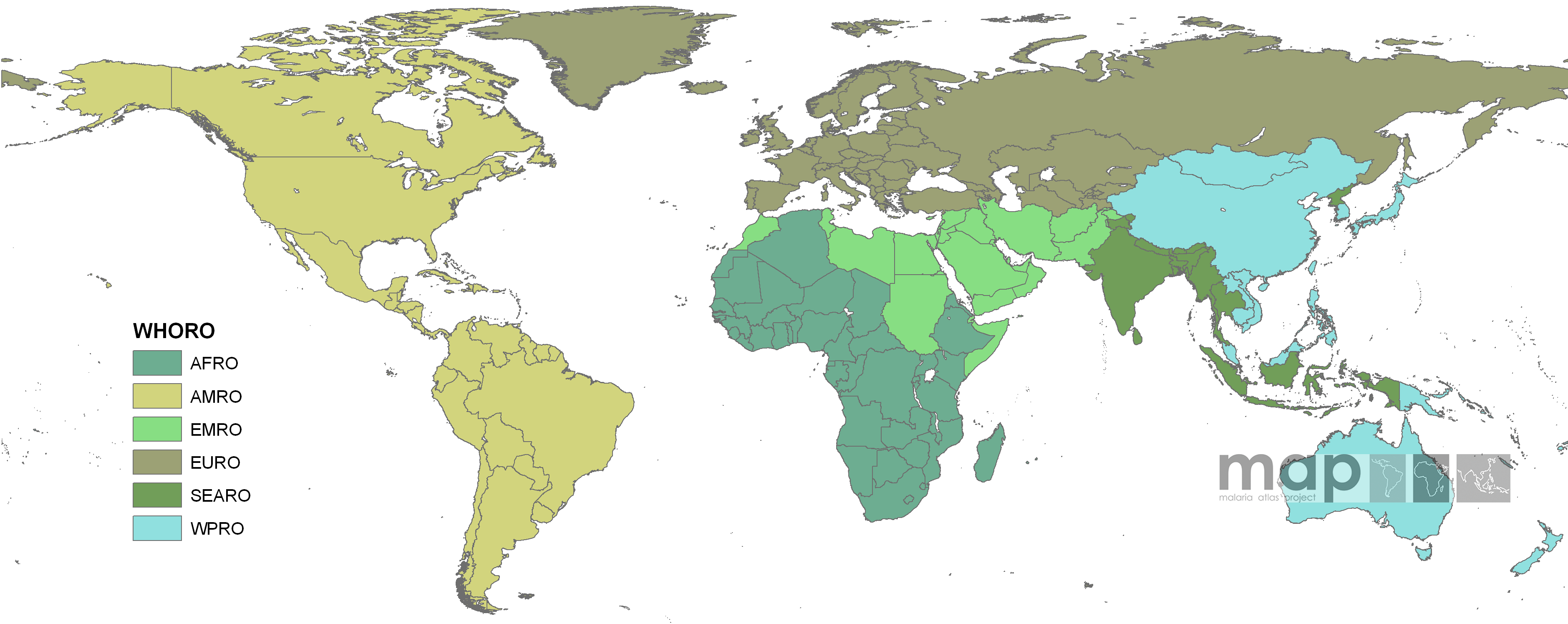

Supplement: Figure S1 — Map of the WHO Regions (http://www.who.int/about/regions/en/index.html). (1.07 MB TIF) [file pmed.1000221.s001.tif]
