# Supplementary material for: Quantifying the Number of Pregnancies at Risk of Malaria in 2007: A Demographic Study
Source: PLoS Med. 2010 Jan 26;7(1):e1000221. doi: 10.1371/journal.pmed.1000221 (PMC2811150; doi:10.1371/journal.pmed.1000221)
Supplement: Table S1 — Demographic characteristics and total population at risk of P. falciparum and/or P. vivax malaria by malaria endemic country and by WHO regional office in 2007 (in millions). (0.38 MB PDF) [file pmed.1000221.s002.pdf]

Table S1: Demographic characteristics and total population at risk of P.falciparum and/or P.vivax malaria by malaria endemic country and by WHO regional office in 2007 (in millions)

2007	Demographic data										Total population at risk*				
	UN National Population Estimates														
	Total Population both sexes*	WOCBAs*	Number of pregnancies*†	TFR	TPR ¥	Pregnancy rate per 1000 WOCBAs	Percentage pregnancies ending in:				*P. falciparum#*			*P. vivax#*	Any species
Countries							Live-births	Still-births	Induced Abortions	Spont. Abortions	Stable¶	Unstable¶	Overall	Overall	Overall
**AFRO**	755	178	35	5.18	7.16	199	72.4%	2.3%	11.9%	13.3%	600	8	608	73	615
Angola [1]	17.02	3.93	0.96	6.43	8.56	245	75.1%	2.5%	9.0%	13.4%	15.41	0.28	15.70	0.00	15.70
Benin [1]	9.03	2.08	0.44	5.42	7.34	210	73.9%	2.3%	10.3%	13.5%	7.66	0.00	7.66	0.00	7.66
Botswana [1]	1.88	0.50	0.06	2.90	4.25	121	68.3%	1.3%	16.4%	14.0%	0.89	0.00	0.89	0.00	0.89
Burkina Faso [1]	14.78	3.39	0.79	6.00	8.12	232	73.9%	2.0%	10.3%	13.8%	14.23	0.00	14.23	0.00	14.23
Burundi [1]	8.51	2.01	0.56	6.80	9.66	276	70.4%	2.5%	14.1%	13.0%	5.66	0.00	5.66	0.00	5.66
Cameroon [1]	18.55	4.47	0.73	4.31	5.74	164	75.1%	2.1%	9.0%	13.8%	16.95	0.00	16.95	0.00	16.95
Cape Verde [1]	0.53	0.14	0.02	3.37	4.56	130	73.9%	1.2%	10.3%	14.6%	0.00	0.25	0.25	0.00	0.25
Central African Republic [1]	4.34	1.03	0.18	4.58	6.10	174	75.1%	2.2%	9.0%	13.7%	4.15	0.00	4.15	0.00	4.15
Chad [1]	10.78	2.41	0.57	6.20	8.26	236	75.1%	2.6%	9.0%	13.3%	9.76	0.16	9.92	0.00	9.92
Comoros [1]	0.84	0.21	0.04	4.30	6.11	174	70.4%	1.9%	14.1%	13.6%	0.59	0.00	0.59	0.64	0.64
Congo [1]	3.77	0.91	0.16	4.49	5.98	171	75.1%	2.2%	9.0%	13.8%	3.33	0.00	3.33	0.00	3.33
Dem. Rep. of the Congo [1]	62.64	13.90	3.55	6.70	8.92	255	75.1%	2.6%	9.0%	13.3%	57.97	0.00	57.97	0.00	57.97
Côte d'Ivoire [1]	19.26	4.58	0.79	4.46	6.04	173	73.9%	2.6%	10.3%	13.2%	17.80	0.00	17.80	0.00	17.80
Equatorial Guinea [1]	0.51	0.12	0.02	5.36	7.14	204	75.1%	2.0%	9.0%	13.9%	0.51	0.00	0.51	0.00	0.51
Eritrea [1]	4.85	1.20	0.25	5.05	7.17	205	70.4%	2.0%	14.1%	13.5%	3.33	0.95	4.28	4.80	4.82
Ethiopia [1]	83.10	19.51	4.19	5.29	7.51	215	70.4%	2.6%	14.1%	12.9%	46.08	1.50	47.59	49.05	53.13
Gabon [1]	1.33	0.35	0.04	3.06	4.08	116	75.1%	1.4%	9.0%	14.5%	1.33	0.00	1.33	0.00	1.33
Gambia [1]	1.71	0.40	0.07	4.70	6.36	182	73.9%	2.1%	10.3%	13.7%	1.51	0.00	1.51	0.00	1.51
Ghana [1]	23.48	5.84	0.87	3.84	5.20	149	73.9%	1.8%	10.3%	14.0%	22.21	0.00	22.21	0.00	22.21
Guinea [1]	9.37	2.14	0.45	5.44	7.37	210	73.9%	2.2%	10.3%	13.6%	9.23	0.00	9.23	0.00	9.23
Guinea-Bissau [1]	1.70	0.37	0.10	7.07	9.57	274	73.9%	2.7%	10.3%	13.1%	1.42	0.00	1.42	0.00	1.42
Kenya [1]	37.54	9.14	1.84	4.96	7.04	201	70.4%	3.3%	14.1%	12.2%	25.62	0.18	25.80	0.00	25.80
Liberia [1]	3.75	0.84	0.22	6.77	9.17	262	73.9%	2.4%	10.3%	13.4%	3.43	0.00	3.43	0.00	3.43
Madagascar [1]	19.68	4.63	0.90	4.78	6.79	194	70.4%	2.1%	14.1%	13.4%	17.28	0.00	17.28	18.74	18.74
Malawi [1]	13.93	3.11	0.70	5.59	7.94	227	70.4%	2.9%	14.1%	12.6%	13.45	0.00	13.45	0.00	13.45
Mali [1]	12.34	2.80	0.71	6.52	8.83	252	73.9%	1.8%	10.3%	14.0%	12.34	0.47	12.34	0.00	12.34
Mauritania [1]	3.12	0.76	0.13	4.37	5.92	169	73.9%	2.3%	10.3%	13.5%	0.93	0.40	1.33	0.00	1.33
Mayotte* [1]	0.21	0.05	0.01	5.65	8.02	229	70.4%	2.3%	14.1%	13.1%	0.00	0.21	0.21	0.00	0.21
Mozambique [1]	21.40	5.10	1.06	5.11	7.26	207	70.4%	2.3%	14.1%	13.2%	21.06	0.00	21.06	0.00	21.06
Namibia [1]	2.07	0.54	0.07	3.19	4.67	133	68.3%	1.3%	16.4%	14.0%	1.25	0.38	1.64	0.00	1.64
Niger [1]	14.23	3.04	0.85	7.19	9.74	278	73.9%	2.9%	10.3%	12.9%	13.19	0.62	13.81	0.00	13.81
Nigeria [1]	148.09	34.57	7.11	5.32	7.20	206	73.9%	2.3%	10.3%	13.5%	134.60	0.00	134.60	0.00	134.60
Rwanda [1]	9.73	2.47	0.59	5.92	8.41	240	70.4%	2.2%	14.1%	13.3%	5.03	0.00	5.03	0.00	5.03
Sao Tome and Principe [1]	0.16	0.04	0.01	3.85	5.13	147	75.1%	1.9%	9.0%	14.0%	0.13	0.00	0.13	0.00	0.13
Senegal [1]	12.38	2.96	0.54	4.69	6.35	181	73.9%	2.0%	10.3%	13.8%	10.82	0.00	10.82	0.00	10.82
Sierra Leone [1]	5.87	1.36	0.34	6.47	8.76	250	73.9%	2.8%	10.3%	13.0%	5.50	0.00	5.50	0.00	5.50

Table S1: Demographic characteristics and total population at risk of P.falciparum and/or P.vivax malaria by malaria endemic country and by WHO regional office in 2007 (in millions)

2007	Demographic data										Total population at risk*				
	UN National Population Estimates														
							Percentage pregnancies ending in:				*P. falciparum#*			*P. vivax#*	Any species
Countries	Total Population both sexes*	WOCBAs*	Number of pregnancies*†	TFR	TPR ¥	Pregnancy rate per 1000 WOCBAs	Live-births	Still-births	Induced Abortions	Spont. Abortions	Stable¶	Unstable¶	Overall	Overall	Overall
South Africa [1]	48.58	12.98	1.43	2.64	3.86	110	68.3%	1.2%	16.4%	14.1%	3.44	2.95	6.39	0.00	6.39
Swaziland [1]	1.14	0.30	0.04	3.45	5.05	144	68.3%	1.5%	16.4%	13.8%	0.23	0.00	0.23	0.00	0.23
Tanzania [1]	40.45	9.38	1.96	5.16	7.33	209	70.4%	2.1%	14.1%	13.4%	39.84	0.00	39.84	0.00	39.84
Togo [1]	6.59	1.58	0.29	4.80	6.50	186	73.9%	2.0%	10.3%	13.8%	5.45	0.00	5.45	0.00	5.45
Uganda [1]	30.88	6.65	1.74	6.46	9.17	262	70.4%	2.3%	14.1%	13.2%	27.03	0.00	27.03	0.00	27.03
Zambia [1]	11.92	2.71	0.57	5.18	7.36	210	70.4%	2.2%	14.1%	13.3%	11.84	0.00	11.84	0.00	11.84
Zimbabwe [1]	13.35	3.42	0.44	3.19	4.53	129	70.4%	1.6%	14.1%	13.9%	7.44	0.00	7.44	0.00	7.44
**EMRO**	409	104	16	3.65	5.31	172	68.8%	2.5%	15.8%	12.8%	90	98	188	262	320
Afghanistan [2]	27.15	5.85	1.73	7.07	10.35	296	68.3%	3.2%	16.4%	12.1%	4.56	12.53	17.10	16.60	20.43
Djibouti [1]	0.83	0.22	0.03	3.95	5.61	160	70.4%	2.5%	14.1%	13.0%	0.02	0.41	0.43	0.71	0.71
Iran [2]	71.21	20.69	1.77	2.04	2.99	85	68.3%	0.7%	16.4%	14.6%	0.15	2.72	2.87	47.46	47.67
Iraq [2]	28.99	7.11	1.25	4.26	6.14	176	69.3%	1.9%	15.3%	13.5%	0.00	0.00	0.00	9.21	9.21
Oman [2]	2.60	0.63	0.08	3.00	4.33	124	69.3%	0.7%	15.3%	14.7%	0.00	0.00	0.00	0.04	0.04
Pakistan [2]	163.90	41.23	6.07	3.52	5.15	147	68.3%	2.9%	16.4%	12.4%	30.74	68.30	99.04	155.73	155.73
Saudi Arabia [2]	24.74	6.00	0.83	3.35	4.83	138	69.3%	0.8%	15.3%	14.6%	0.72	1.22	1.94	13.00	14.39
Somalia [1]	8.70	2.04	0.50	6.04	8.58	245	70.4%	3.3%	14.1%	12.2%	8.70	0.55	8.70	8.70	8.70
Sudan [1]	38.56	9.35	1.62	4.23	6.05	173	69.9%	3.9%	14.7%	11.5%	28.99	6.84	35.83	2.98	35.93
Syrian Arab Rep. [2]	19.93	5.35	0.68	3.08	4.44	127	69.3%	0.8%	15.3%	14.6%	0.00	0.00	0.00	5.88	5.88
Yemen [2]	22.39	5.18	1.17	5.50	7.93	227	69.3%	2.5%	15.3%	12.9%	15.93	5.72	21.65	1.88	21.74
**EURO**	135	37	3	2.23	3.24	91	68.9%	1.1%	15.7%	14.2%	0	3	3	23	23
Armenia [6]	3.00	0.87	0.05	1.39	2.00	57	69.3%	0.8%	15.3%	14.6%	0.00	0.00	0.00	0.35	0.35
Azerbaijan [6]	8.47	2.56	0.19	1.82	2.62	75	69.3%	0.4%	15.3%	15.0%	0.00	0.00	0.00	0.22	0.22
Georgia [6]	4.40	1.17	0.07	1.41	2.03	58	69.3%	1.0%	15.3%	14.4%	0.00	0.00	0.00	0.69	0.69
Kyrgyzstan [2]	5.32	1.47	0.15	2.48	3.63	104	68.3%	1.3%	16.4%	14.0%	0.00	1.20	1.20	1.62	1.66
Tajikistan [2]	6.74	1.78	0.25	3.35	4.90	140	68.3%	1.7%	16.4%	13.6%	0.00	2.16	2.16	4.41	4.87
Turkey [6]	74.88	20.51	1.81	2.14	3.09	88	69.3%	1.0%	15.3%	14.4%	0.00	0.00	0.00	13.99	13.99
Turkmenistan [2]	4.97	1.42	0.15	2.50	3.66	105	68.3%	1.3%	16.4%	14.0%	0.00	0.00	0.00	1.35	1.35
Uzbekistan [2]	27.37	0.01	0.79	2.49	3.65	104	68.3%	1.3%	16.4%	14.0%	0.00	0.00	0.00	0.29	0.29
**AMRO**	530	143	16	2.41	3.81	124	63.2%	0.9%	22.0%	14.0%	41	50	91	96	138
Argentina [5]	39.53	9.95	1.03	2.25	3.64	104	61.8%	0.7%	23.5%	14.0%	0.00	0.00	0.00	3.02	3.02
Belize [4]	0.29	0.07	0.01	2.93	4.35	124	67.3%	1.1%	17.5%	14.1%	0.00	0.17	0.17	0.25	0.26
Bolivia [5]	9.53	2.37	0.38	3.50	5.66	162	61.8%	1.3%	23.5%	13.4%	0.22	2.61	2.83	4.05	4.06
Brazil [5]	191.79	52.64	5.48	2.25	3.64	104	61.8%	0.8%	23.5%	13.9%	12.79	16.69	29.47	23.07	34.48
Colombia [5]	46.16	12.71	1.30	2.22	3.59	103	61.8%	0.8%	23.5%	13.9%	5.26	7.74	13.00	17.32	21.44

Table S1: Demographic characteristics and total population at risk of P.falciparum and/or P.vivax malaria by malaria endemic country and by WHO regional office in 2007 (in millions)

2007	Demographic data										Total population at risk*				
	UN National Population Estimates														
							Percentage pregnancies ending in:				*P. falciparum*#			*P. vivax*#	Any species
Countries	Total Population both sexes*	WOCBAs*	Number of pregnancies*†	TFR	TPR ¥	Pregnancy rate per 1000 WOCBAs	Live-births	Still-births	Induced Abortions	Spont. Abortions	Stable¶	Unstable¶	Overall	Overall	Overall
Costa Rica [4]	4.47	1.21	0.11	2.10	3.12	89	67.3%	0.7%	17.5%	14.5%	0.00	0.00	0.00	1.03	1.03
Dominican Republic [4]	9.76	2.52	0.34	2.81	4.67	133	60.2%	0.9%	25.3%	13.7%	1.41	2.83	4.24	0.00	4.24
Ecuador [5]	13.34	3.44	0.41	2.58	4.17	119	61.8%	1.0%	23.5%	13.7%	4.12	1.65	5.77	3.73	5.85
El Salvador [4]	6.86	1.82	0.21	2.68	3.98	114	67.3%	1.1%	17.5%	14.1%	0.00	0.00	0.00	1.10	1.10
Guatemala [4]	13.35	3.24	0.01	3.27	5.29	151	61.8%	0.8%	23.5%	13.9%	0.14	0.00	0.14	0.20	0.20
Guyana [5]	0.74	0.18	0.57	4.15	6.17	176	67.3%	2.3%	17.5%	12.9%	1.02	5.27	6.30	4.07	7.30
Haiti [4]	9.60	2.47	0.02	2.33	3.77	108	61.8%	1.0%	23.5%	13.7%	0.14	0.52	0.66	0.74	0.74
Honduras [4]	7.11	1.81	0.42	3.54	5.88	168	60.2%	1.5%	25.3%	13.0%	8.60	0.00	8.60	0.00	8.60
Mexico [4]	106.54	29.58	0.25	3.31	4.92	141	67.3%	1.3%	17.5%	13.9%	0.88	2.63	3.51	5.46	6.47
Nicaragua [4]	5.60	1.48	2.78	2.21	3.28	94	67.3%	0.5%	17.5%	14.7%	0.00	0.00	0.00	15.82	15.82
Panama [4]	3.34	0.88	0.17	2.76	4.10	117	67.3%	1.3%	17.5%	13.9%	1.57	2.12	3.70	2.21	4.71
Paraguay [5]	6.13	1.55	0.10	2.56	3.80	109	67.3%	0.9%	17.5%	14.3%	0.90	0.00	0.90	0.19	1.06
Peru [5]	27.90	7.45	0.22	3.08	4.98	142	61.8%	1.0%	23.5%	13.7%	0.00	0.00	0.00	1.52	1.52
Suriname [5]	0.46	0.12	0.86	2.51	4.06	116	61.8%	0.9%	23.5%	13.8%	3.87	1.70	5.57	8.14	8.33
Venezuela [5]	27.66	7.33	0.01	2.42	3.92	112	61.8%	0.9%	23.5%	13.8%	0.01	0.05	0.06	0.04	0.06
French Guiana [5]	0.20	0.05	0.86	2.55	4.13	118	61.8%	0.8%	23.5%	13.9%	0.22	6.24	6.46	4.29	7.89
**SEARO**	1,770	458	52	2.62	3.94	122	66.3%	1.9%	18.5%	13.2%	564	712	1,276	1,632	1,639
Bangladesh [2]	158.67	41.02	4.86	2.83	4.14	118	68.3%	2.7%	16.4%	12.6%	15.12	47.99	63.11	154.08	154.08
Bhutan [2]	0.66	0.17	0.02	2.19	3.21	92	68.3%	1.7%	16.4%	13.6%	0.66	0.47	0.66	0.66	0.66
Burma [2]	48.80	13.97	1.40	2.07	3.51	100	59.0%	1.3%	26.5%	13.2%	42.88	1.91	44.79	47.47	48.80
India [2]	1,169.02	294.81	34.65	2.81	4.11	118	68.3%	2.1%	16.4%	13.2%	414.53	535.59	950.12	1109.03	1109.03
Indonesia [2]	231.63	64.13	6.77	2.18	3.70	106	59.0%	0.7%	26.5%	13.7%	68.59	81.93	150.52	215.66	217.60
Republic of Korea [2]	48.22	13.10	0.80	1.21	2.13	61	56.8%	1.0%	29.0%	13.2%	0.00	0.00	0.00	12.39	12.39
Nepal [2]	28.20	7.18	0.98	3.28	4.80	137	68.3%	3.9%	16.4%	11.4%	3.40	6.15	9.54	18.01	18.92
Sri Lanka [2]	19.30	5.29	0.42	1.88	2.75	79	68.3%	0.7%	16.4%	14.6%	1.75	7.53	9.28	10.46	12.84
Thailand [2]	63.88	17.85	1.60	1.85	3.14	90	59.0%	0.6%	26.5%	13.9%	16.53	30.53	47.06	63.88	63.88
Timor-Leste [2]	1.16	0.26	0.08	6.53	11.07	316	59.0%	0.8%	26.5%	13.7%	0.96	0.00	0.96	0.63	0.96
**WPRO**	1,558	423	40	1.88	3.27	139	57.3%	1.3%	28.4%	13.0%	91	113	203	1,090	1,131
Cambodia [2]	14.44	3.87	0.60	3.18	5.39	154	59.0%	1.2%	26.5%	13.2%	10.77	2.55	13.33	12.65	14.44
China [2]	1,328.63	361.61	31.48	1.73	3.05	87	56.8%	1.4%	29.0%	12.9%	17.13	20.32	37.45	896.81	915.87
Lao [2]	5.86	1.52	0.24	3.21	5.44	155	59.0%	1.2%	26.5%	13.2%	5.30	0.01	5.31	2.32	5.32
Malaysia [2]	26.57	7.06	0.89	2.60	4.41	126	59.0%	0.5%	26.5%	14.0%	6.29	16.17	22.46	10.15	22.66
Papua New Guinea [3]	6.33	1.60	0.25	3.78	5.45	156	69.3%	1.2%	15.3%	14.2%	4.11	0.00	4.11	3.90	4.46
Philippines [2]	87.96	22.52	3.52	3.23	5.47	156	59.0%	0.7%	26.5%	13.7%	26.95	20.41	47.35	78.55	82.01

Table S1: Demographic characteristics and total population at risk of P.falciparum and/or P.vivax malaria by malaria endemic country and by WHO regional office in 2007 (in millions)

2007	Demographic data										Total population at risk*				
	UN National Population Estimates														
	Total Population both sexes*	WOCBAs*	Number of pregnancies*†	TFR	TPR ¥	Pregnancy rate per 1000 WOCBAs	Percentage pregnancies ending in:				*P. falciparum*#			*P. vivax*#	Any species
Countries							Live-births	Still-births	Induced Abortions	Spont. Abortions	Stable¶	Unstable¶	Overall	Overall	Overall
Solomon Islands [3]	0.50	0.12	0.02	3.87	5.58	159	69.3%	0.9%	15.3%	14.4%	0.43	0.00	0.43	0.38	0.49
Vanuatu [3]	0.23	0.06	0.01	3.74	5.39	154	69.3%	1.0%	15.3%	14.4%	0.22	0.00	0.22	0.20	0.22
Viet Nam [2]	87.38	24.82	2.57	2.14	3.63	104	59.0%	0.7%	26.5%	13.8%	19.31	53.31	72.62	85.08	85.49
**TOTAL**	**5,157**	**1,343**	**162**	**2.77**	**4.23**	**159**	**65.5%**	**1.8%**	**19.5%**	**13.3%**	**1,386**	**985**	**2,369**	**3,177**	**3,868**

* In millions

† The total number of pregnancies is the sum of the number of live-births, stillbirths, miscarriages and induced abortions.

¥ TPR is the total number of pregnancies during the period of child-bearing age of a women (15 to 49 years of age).

Includes countries where *P. falciparum* and *P. vivax* co-exist

¶ Stable transmission: >= 0.1 autochthonous P. falciparum cases per 1,000 people per annum; unstable transmission <0.1 autochthonous P. falciparum cases per 1,000 people per annum

Abbreviation: TFR: Total Fertility Rate; TPR: Total Pregnancy Rate; UN: United Nations; WOCBA: Women of Childbearing Age (15-49 years of age); PWAR: Pregnant Women at Risk

Note: The regional and total estimates for TFR, Stillbirth rate, TPR and pregnancy rates are weighted means. The regional and total numbers at risk was derived directly as the sum of the national estimates within each region and globally.

Continents: The number in superscript after each country's name refers to the continent to which the country belongs: 1 = Africa; 2=Asia; 3=Oceania; 4=North America; 5=South America; 6=Europe
