# Supplementary material for: Quantifying the Number of Pregnancies at Risk of Malaria in 2007: A Demographic Study
Source: PLoS Med. 2010 Jan 26;7(1):e1000221. doi: 10.1371/journal.pmed.1000221 (PMC2811150; doi:10.1371/journal.pmed.1000221)
Supplement: Table S2 — Total number of pregnancies by pregnancy outcome in areas with P. falciparum and/or P. vivax transmission by continent in 2007 (in millions). (0.54 MB PDF) [file pmed.1000221.s003.pdf]

Table S2: Total number of pregnancies by pregnancy outcome in areas with P. falciparum and/or P. vivax transmission by continent in 2007 (in millions)

**2007**	**Number of pregnancies at risk of malaria*†**					**Number of live-births born to pregnancies at risk of malaria* †**					**Number of stillbirths born to pregnancies at risk of malaria* †**					**Number of miscarriages among pregnancies at risk of malaria* †**					**Number of induced abortions among pregnancies at risk of malaria* †**				
	P. falciparum#			*P. vivax#*	Any species	*P. falciparum#*			*P. vivax#*	Any species	*P. falciparum#*			*P. vivax#*	Any species	*P. falciparum#*			*P. vivax#*	Any species	*P. falciparum#*			*P. vivax#*	Any species
**Countries**	Stable¶	Unstable¶	Overall	Overall	Overall	Stable¶	Unstable¶	Overall	Overall	Overall	Stable¶	Unstable¶	Overall	Overall	Overall	Stable¶	Unstable¶	Overall	Overall	Overall	Stable¶	Unstable¶	Overall	Overall	Overall
**AFRO**	29.624	0.357	29.954	3.598	30.330	21.553	0.254	21.787	2.533	22.051	0.703	0.007	0.710	0.088	0.719	3.950	0.048	3.994	0.469	4.043	3.419	0.048	3.464	0.507	3.517
Angola [1]	0.871	0.016	0.887	0.000	0.887	0.654	0.012	0.666	0.000	0.666	0.022	0.000	0.022	0.000	0.022	0.117	0.002	0.119	0.000	0.119	0.078	0.001	0.080	0.000	0.080
Benin [1]	0.369	0.000	0.369	0.000	0.369	0.273	0.000	0.273	0.000	0.273	0.008	0.000	0.008	0.000	0.008	0.050	0.000	0.050	0.000	0.050	0.038	0.000	0.038	0.000	0.038
Botswana [1]	0.029	0.000	0.029	0.000	0.029	0.020	0.000	0.020	0.000	0.020	0.000	0.000	0.000	0.000	0.000	0.004	0.000	0.004	0.000	0.004	0.005	0.000	0.005	0.000	0.005
Burkina Faso [1]	0.758	0.000	0.758	0.000	0.758	0.560	0.000	0.560	0.000	0.560	0.015	0.000	0.015	0.000	0.015	0.105	0.000	0.105	0.000	0.105	0.078	0.000	0.078	0.000	0.078
Burundi [1]	0.370	0.000	0.370	0.000	0.370	0.260	0.000	0.260	0.000	0.260	0.009	0.000	0.009	0.000	0.009	0.048	0.000	0.048	0.000	0.048	0.052	0.000	0.052	0.000	0.052
Cameroon [1]	0.670	0.000	0.670	0.000	0.670	0.503	0.000	0.503	0.000	0.503	0.014	0.000	0.014	0.000	0.014	0.093	0.000	0.093	0.000	0.093	0.060	0.000	0.060	0.000	0.060
Cape Verde [1]	0.000	0.009	0.009	0.000	0.009	0.000	0.006	0.006	0.000	0.006	0.000	0.000	0.000	0.000	0.000	0.000	0.001	0.001	0.000	0.001	0.000	0.001	0.001	0.000	0.001
Central African Republic [1]	0.172	0.000	0.172	0.000	0.172	0.129	0.000	0.129	0.000	0.129	0.004	0.000	0.004	0.000	0.004	0.024	0.000	0.024	0.000	0.024	0.016	0.000	0.016	0.000	0.016
Chad [1]	0.515	0.009	0.523	0.000	0.523	0.387	0.006	0.393	0.000	0.393	0.014	0.000	0.014	0.000	0.014	0.068	0.001	0.070	0.000	0.070	0.046	0.001	0.047	0.000	0.047
Comoros [1]	0.025	0.000	0.025	0.027	0.027	0.018	0.000	0.018	0.019	0.019	0.000	0.000	0.000	0.001	0.001	0.003	0.000	0.003	0.004	0.004	0.004	0.000	0.004	0.004	0.004
Congo [1]	0.137	0.000	0.137	0.000	0.137	0.103	0.000	0.103	0.000	0.103	0.003	0.000	0.003	0.000	0.003	0.019	0.000	0.019	0.000	0.019	0.012	0.000	0.012	0.000	0.012
Dem. Rep. of the Congo [1]	3.281	0.000	3.281	0.000	3.281	2.463	0.000	2.463	0.000	2.463	0.086	0.000	0.086	0.000	0.086	0.436	0.000	0.436	0.000	0.436	0.296	0.000	0.296	0.000	0.296
Côte d'Ivoire [1]	0.729	0.000	0.729	0.000	0.729	0.539	0.000	0.539	0.000	0.539	0.019	0.000	0.019	0.000	0.019	0.096	0.000	0.096	0.000	0.096	0.075	0.000	0.075	0.000	0.075
Equatorial Guinea [1]	0.024	0.000	0.024	0.000	0.024	0.018	0.000	0.018	0.000	0.018	0.000	0.000	0.000	0.000	0.000	0.003	0.000	0.003	0.000	0.003	0.002	0.000	0.002	0.000	0.002
Eritrea [1]	0.168	0.048	0.217	0.243	0.244	0.119	0.034	0.153	0.171	0.172	0.003	0.001	0.004	0.005	0.005	0.023	0.007	0.029	0.033	0.033	0.024	0.007	0.031	0.034	0.034
Ethiopia [1]	2.322	0.076	2.397	2.471	2.676	1.635	0.053	1.688	1.740	1.885	0.061	0.002	0.063	0.065	0.071	0.298	0.010	0.308	0.318	0.344	0.327	0.011	0.338	0.348	0.377
Gabon [1]	0.040	0.000	0.040	0.000	0.040	0.030	0.000	0.030	0.000	0.030	0.001	0.000	0.001	0.000	0.001	0.006	0.000	0.006	0.000	0.006	0.004	0.000	0.004	0.000	0.004
Gambia [1]	0.065	0.000	0.065	0.000	0.065	0.048	0.000	0.048	0.000	0.048	0.001	0.000	0.001	0.000	0.001	0.009	0.000	0.009	0.000	0.009	0.007	0.000	0.007	0.000	0.007
Ghana [1]	0.821	0.000	0.821	0.000	0.821	0.606	0.000	0.606	0.000	0.606	0.015	0.000	0.015	0.000	0.015	0.115	0.000	0.115	0.000	0.115	0.085	0.000	0.085	0.000	0.085
Guinea [1]	0.444	0.000	0.444	0.000	0.444	0.328	0.000	0.328	0.000	0.328	0.010	0.000	0.010	0.000	0.010	0.061	0.000	0.061	0.000	0.061	0.046	0.000	0.046	0.000	0.046
Guinea-Bissau [1]	0.085	0.000	0.085	0.000	0.085	0.063	0.000	0.063	0.000	0.063	0.002	0.000	0.002	0.000	0.002	0.011	0.000	0.011	0.000	0.011	0.009	0.000	0.009	0.000	0.009
Kenya [1]	1.255	0.009	1.264	0.000	1.264	0.884	0.006	0.890	0.000	0.890	0.041	0.000	0.041	0.000	0.041	0.153	0.001	0.154	0.000	0.154	0.177	0.001	0.178	0.000	0.178
Liberia [1]	0.200	0.000	0.200	0.000	0.200	0.148	0.000	0.148	0.000	0.148	0.005	0.000	0.005	0.000	0.005	0.027	0.000	0.027	0.000	0.027	0.021	0.000	0.021	0.000	0.021
Madagascar [1]	0.789	0.000	0.789	0.856	0.856	0.556	0.000	0.556	0.603	0.603	0.016	0.000	0.016	0.018	0.018	0.106	0.000	0.106	0.115	0.115	0.111	0.000	0.111	0.121	0.121
Malawi [1]	0.680	0.000	0.680	0.000	0.680	0.479	0.000	0.479	0.000	0.479	0.019	0.000	0.019	0.000	0.019	0.086	0.000	0.086	0.000	0.086	0.096	0.000	0.096	0.000	0.096
Mali [1]	0.705	0.027	0.705	0.000	0.705	0.521	0.020	0.521	0.000	0.521	0.013	0.000	0.013	0.000	0.013	0.099	0.004	0.099	0.000	0.099	0.073	0.003	0.073	0.000	0.073
Mauritania [1]	0.038	0.017	0.055	0.000	0.055	0.028	0.012	0.040	0.000	0.040	0.001	0.000	0.001	0.000	0.001	0.005	0.002	0.007	0.000	0.007	0.004	0.002	0.006	0.000	0.006
Mayotte* [1]	0.000	0.010	0.010	0.000	0.010	0.000	0.007	0.007	0.000	0.007	0.000	0.000	0.000	0.000	0.000	0.000	0.001	0.001	0.000	0.001	0.000	0.001	0.001	0.000	0.001
Mozambique [1]	1.041	0.000	1.041	0.000	1.041	0.733	0.000	0.733	0.000	0.733	0.024	0.000	0.024	0.000	0.024	0.137	0.000	0.137	0.000	0.137	0.147	0.000	0.147	0.000	0.147
Namibia [1]	0.044	0.013	0.057	0.000	0.057	0.030	0.009	0.039	0.000	0.039	0.001	0.000	0.001	0.000	0.001	0.006	0.002	0.008	0.000	0.008	0.007	0.002	0.009	0.000	0.009
Niger [1]	0.785	0.037	0.822	0.000	0.822	0.580	0.027	0.607	0.000	0.607	0.023	0.001	0.024	0.000	0.024	0.101	0.005	0.106	0.000	0.106	0.081	0.004	0.085	0.000	0.085
Nigeria [1]	6.466	0.000	6.466	0.000	6.466	4.775	0.000	4.775	0.000	4.775	0.147	0.000	0.147	0.000	0.147	0.875	0.000	0.875	0.000	0.875	0.669	0.000	0.669	0.000	0.669
Rwanda [1]	0.307	0.000	0.307	0.000	0.307	0.216	0.000	0.216	0.000	0.216	0.007	0.000	0.007	0.000	0.007	0.041	0.000	0.041	0.000	0.041	0.043	0.000	0.043	0.000	0.043
Sao Tome and Principe [1]	0.005	0.000	0.005	0.000	0.005	0.004	0.000	0.004	0.000	0.004	0.000	0.000	0.000	0.000	0.000	0.001	0.000	0.001	0.000	0.001	0.000	0.000	0.000	0.000	0.000
Senegal [1]	0.470	0.000	0.470	0.000	0.470	0.347	0.000	0.347	0.000	0.347	0.010	0.000	0.010	0.000	0.010	0.065	0.000	0.065	0.000	0.065	0.049	0.000	0.049	0.000	0.049
Sierra Leone [1]	0.320	0.000	0.320	0.000	0.320	0.236	0.000	0.236	0.000	0.236	0.009	0.000	0.009	0.000	0.009	0.042	0.000	0.042	0.000	0.042	0.033	0.000	0.033	0.000	0.033
South Africa [1]	0.101	0.087	0.189	0.000	0.189	0.069	0.060	0.129	0.000	0.129	0.001	0.001	0.002	0.000	0.002	0.014	0.012	0.027	0.000	0.027	0.017	0.014	0.031	0.000	0.031
Swaziland [1]	0.009	0.000	0.009	0.000	0.009	0.006	0.000	0.006	0.000	0.006	0.000	0.000	0.000	0.000	0.000	0.001	0.000	0.001	0.000	0.001	0.001	0.000	0.001	0.000	0.001
Tanzania [1]	1.934	0.000	1.934	0.000	1.934	1.362	0.000	1.362	0.000	1.362	0.041	0.000	0.041	0.000	0.041	0.258	0.000	0.258	0.000	0.258	0.272	0.000	0.272	0.000	0.272
Togo [1]	0.242	0.000	0.242	0.000	0.242	0.179	0.000	0.179	0.000	0.179	0.005	0.000	0.005	0.000	0.005	0.033	0.000	0.033	0.000	0.033	0.025	0.000	0.025	0.000	0.025
Uganda [1]	1.526	0.000	1.526	0.000	1.526	1.074	0.000	1.074	0.000	1.074	0.036	0.000	0.036	0.000	0.036	0.201	0.000	0.201	0.000	0.201	0.215	0.000	0.215	0.000	0.215
Zambia [1]	0.566	0.000	0.566	0.000	0.566	0.399	0.000	0.399	0.000	0.399	0.013	0.000	0.013	0.000	0.013	0.075	0.000	0.075	0.000	0.075	0.080	0.000	0.080	0.000	0.080
Zimbabwe [1]	0.247	0.000	0.247	0.000	0.247	0.174	0.000	0.174	0.000	0.174	0.004	0.000	0.004	0.000	0.004	0.034	0.000	0.034	0.000	0.034	0.035	0.000	0.035	0.000	0.035
**EMRO**	4.009	4.072	8.050	9.790	12.509	2.777	2.791	5.546	6.712	8.602	0.128	0.121	0.248	0.250	0.338	0.489	0.503	0.988	1.250	1.581	0.615	0.658	1.268	1.578	1.987
Afghanistan [2]	0.291	0.799	1.090	1.058	1.303	0.199	0.546	0.745	0.723	0.890	0.009	0.026	0.035	0.034	0.042	0.035	0.097	0.132	0.128	0.158	0.048	0.131	0.179	0.173	0.214
Djibouti [1]	0.001	0.017	0.018	0.029	0.029	0.001	0.012	0.012	0.021	0.021	0.000	0.000	0.000	0.001	0.001	0.000	0.002	0.002	0.004	0.004	0.000	0.002	0.002	0.004	0.004
Iran [2]	0.004	0.068	0.071	1.177	1.182	0.002	0.046	0.049	0.804	0.807	0.000	0.001	0.001	0.009	0.009	0.001	0.010	0.010	0.171	0.172	0.001	0.011	0.012	0.193	0.194
Iraq [2]	0.000	0.000	0.000	0.396	0.396	0.000	0.000	0.000	0.275	0.275	0.000	0.000	0.000	0.008	0.008	0.000	0.000	0.000	0.053	0.053	0.000	0.000	0.000	0.060	0.060
Oman [2]	0.000	0.000	0.000	0.001	0.001	0.000	0.000	0.000	0.001	0.001	0.000	0.000	0.000	0.000	0.000	0.000	0.000	0.000	0.000	0.000	0.000	0.000	0.000	0.000	0.000
Pakistan [2]	1.138	2.530	3.668	5.767	5.767	0.778	1.728	2.505	3.940	3.940	0.034	0.075	0.108	0.170	0.170	0.141	0.312	0.453	0.712	0.712	0.187	0.415	0.601	0.945	0.945

Table S2: Total number of pregnancies by pregnancy outcome in areas with P. falciparum and/or P. vivax transmission by continent in 2007 (in millions)

2007	Number of pregnancies at risk of malaria*†					Number of live-births born to pregnancies at risk of malaria* †					Number of stillbirths born to pregnancies at risk of malaria* †					Number of miscarriages among pregnancies at risk of malaria* †					Number of induced abortions among pregnancies at risk of malaria* †				
	P. falciparum#			*P. vivax#*	Any species	*P. falciparum#*			*P. vivax#*	Any species	*P. falciparum#*			*P. vivax#*	Any species	*P. falciparum#*			*P. vivax#*	Any species	*P. falciparum#*			*P. vivax#*	Any species
**Countries**	Stable¶	Unstable¶	Overall	Overall	Overall	Stable¶	Unstable¶	Overall	Overall	Overall	Stable¶	Unstable¶	Overall	Overall	Overall	Stable¶	Unstable¶	Overall	Overall	Overall	Stable¶	Unstable¶	Overall	Overall	Overall
Saudi Arabia [2]	0.024	0.041	0.065	0.435	0.482	0.017	0.028	0.045	0.302	0.334	0.000	0.000	0.000	0.003	0.004	0.004	0.006	0.010	0.064	0.071	0.004	0.006	0.010	0.066	0.074
Somalia [1]	0.501	0.031	0.501	0.501	0.501	0.353	0.022	0.353	0.353	0.353	0.017	0.001	0.017	0.017	0.017	0.061	0.004	0.061	0.061	0.061	0.071	0.004	0.071	0.071	0.071
Sudan [1]	1.216	0.287	1.503	0.125	1.507	0.850	0.201	1.050	0.087	1.053	0.048	0.011	0.059	0.005	0.059	0.140	0.033	0.173	0.014	0.174	0.178	0.042	0.221	0.018	0.221
Syrian Arab Rep. [2]	0.000	0.000	0.000	0.200	0.200	0.000	0.000	0.000	0.139	0.139	0.000	0.000	0.000	0.002	0.002	0.000	0.000	0.000	0.029	0.029	0.000	0.000	0.000	0.031	0.031
Yemen [2]	0.835	0.300	1.135	0.099	1.139	0.579	0.208	0.787	0.068	0.790	0.021	0.007	0.028	0.002	0.028	0.108	0.039	0.147	0.013	0.147	0.127	0.046	0.173	0.015	0.174
**EURO**	0.000	0.114	0.114	0.618	0.636	0.000	0.078	0.078	0.426	0.438	0.000	0.002	0.002	0.008	0.008	0.000	0.016	0.016	0.087	0.090	0.000	0.019	0.019	0.097	0.100
Armenia [6]	0.000	0.000	0.000	0.006	0.006	0.000	0.000	0.000	0.004	0.004	0.000	0.000	0.000	0.000	0.000	0.000	0.000	0.000	0.001	0.001	0.000	0.000	0.000	0.001	0.001
Azerbaijan [6]	0.000	0.000	0.000	0.005	0.005	0.000	0.000	0.000	0.004	0.004	0.000	0.000	0.000	0.000	0.000	0.000	0.000	0.000	0.001	0.001	0.000	0.000	0.000	0.001	0.001
Georgia [6]	0.000	0.000	0.000	0.011	0.011	0.000	0.000	0.000	0.007	0.007	0.000	0.000	0.000	0.000	0.000	0.000	0.000	0.000	0.002	0.002	0.000	0.000	0.000	0.002	0.002
Kyrgyzstan [2]	0.000	0.034	0.034	0.047	0.048	0.000	0.024	0.024	0.032	0.033	0.000	0.000	0.000	0.001	0.001	0.000	0.005	0.005	0.007	0.007	0.000	0.006	0.006	0.008	0.008
Tajikistan [2]	0.000	0.080	0.080	0.163	0.180	0.000	0.055	0.055	0.111	0.123	0.000	0.001	0.001	0.003	0.003	0.000	0.011	0.011	0.022	0.024	0.000	0.013	0.013	0.027	0.030
Turkey [6]	0.000	0.000	0.000	0.338	0.338	0.000	0.000	0.000	0.234	0.234	0.000	0.000	0.000	0.003	0.003	0.000	0.000	0.000	0.049	0.049	0.000	0.000	0.000	0.052	0.052
Turkmenistan [2]	0.000	0.000	0.000	0.040	0.040	0.000	0.000	0.000	0.028	0.028	0.000	0.000	0.000	0.001	0.001	0.000	0.000	0.000	0.006	0.006	0.000	0.000	0.000	0.007	0.007
Uzbekistan [2]	0.000	0.000	0.000	0.008	0.008	0.000	0.000	0.000	0.006	0.006	0.000	0.000	0.000	0.000	0.000	0.000	0.000	0.000	0.001	0.001	0.000	0.000	0.000	0.001	0.001
**AMRO**	1.356	1.602	2.958	2.895	4.318	0.840	1.010	1.849	1.840	2.726	0.015	0.018	0.033	0.028	0.045	0.184	0.219	0.404	0.403	0.596	0.317	0.355	0.672	0.625	0.952
Argentina [5]	0.000	0.000	0.000	0.079	0.079	0.000	0.000	0.000	0.049	0.049	0.000	0.000	0.000	0.001	0.001	0.000	0.000	0.000	0.011	0.011	0.000	0.000	0.000	0.019	0.019
Belize [4]	0.000	0.006	0.006	0.008	0.008	0.000	0.004	0.004	0.005	0.006	0.000	0.000	0.000	0.000	0.000	0.000	0.001	0.001	0.001	0.001	0.000	0.001	0.001	0.001	0.001
Bolivia [5]	0.009	0.105	0.114	0.163	0.164	0.006	0.065	0.070	0.101	0.101	0.000	0.001	0.002	0.002	0.002	0.001	0.014	0.015	0.022	0.022	0.002	0.025	0.027	0.038	0.038
Brazil [5]	0.365	0.476	0.841	0.659	0.984	0.226	0.294	0.520	0.407	0.608	0.003	0.004	0.007	0.005	0.008	0.051	0.066	0.117	0.091	0.137	0.086	0.112	0.198	0.155	0.231
Colombia [5]	0.149	0.219	0.367	0.489	0.606	0.092	0.135	0.227	0.302	0.374	0.001	0.002	0.003	0.004	0.005	0.021	0.030	0.051	0.068	0.084	0.035	0.051	0.086	0.115	0.142
Costa Rica [4]	0.000	0.000	0.000	0.025	0.025	0.000	0.000	0.000	0.017	0.017	0.000	0.000	0.000	0.000	0.000	0.000	0.000	0.000	0.004	0.004	0.000	0.000	0.000	0.004	0.004
Dominican Republic [4]	0.049	0.098	0.146	0.000	0.146	0.029	0.059	0.088	0.000	0.088	0.000	0.001	0.001	0.000	0.001	0.007	0.013	0.020	0.000	0.020	0.012	0.025	0.037	0.000	0.037
Ecuador [5]	0.127	0.051	0.177	0.115	0.180	0.078	0.031	0.109	0.071	0.111	0.001	0.000	0.002	0.001	0.002	0.017	0.007	0.024	0.016	0.025	0.030	0.012	0.042	0.027	0.042
El Salvador [4]	0.000	0.000	0.000	0.033	0.033	0.000	0.000	0.000	0.022	0.022	0.000	0.000	0.000	0.000	0.000	0.000	0.000	0.000	0.005	0.005	0.000	0.000	0.000	0.006	0.006
Guatemala [4]	0.005	0.000	0.005	0.007	0.007	0.003	0.000	0.003	0.005	0.005	0.000	0.000	0.000	0.000	0.000	0.001	0.000	0.001	0.001	0.001	0.001	0.000	0.001	0.002	0.002
Guyana [5]	0.044	0.225	0.269	0.174	0.312	0.029	0.152	0.181	0.117	0.210	0.001	0.005	0.006	0.004	0.007	0.006	0.029	0.035	0.022	0.040	0.008	0.039	0.047	0.030	0.055
Haiti [4]	0.004	0.014	0.017	0.019	0.019	0.002	0.008	0.011	0.012	0.012	0.000	0.000	0.000	0.000	0.000	0.001	0.002	0.002	0.003	0.003	0.001	0.003	0.004	0.005	0.005
Honduras [4]	0.372	0.000	0.372	0.000	0.372	0.224	0.000	0.224	0.000	0.224	0.006	0.000	0.006	0.000	0.006	0.049	0.000	0.049	0.000	0.049	0.094	0.000	0.094	0.000	0.094
Mexico [4]	0.032	0.094	0.126	0.195	0.231	0.021	0.063	0.085	0.131	0.156	0.000	0.001	0.002	0.002	0.003	0.004	0.013	0.018	0.027	0.032	0.006	0.016	0.022	0.034	0.040
Nicaragua [4]	0.000	0.000	0.000	0.412	0.412	0.000	0.000	0.000	0.277	0.277	0.000	0.000	0.000	0.002	0.002	0.000	0.000	0.000	0.061	0.061	0.000	0.000	0.000	0.072	0.072
Panama [4]	0.049	0.066	0.115	0.068	0.146	0.033	0.044	0.077	0.046	0.098	0.001	0.001	0.001	0.001	0.002	0.007	0.009	0.016	0.010	0.020	0.009	0.012	0.020	0.012	0.026
Paraguay [5]	0.026	0.000	0.026	0.005	0.030	0.017	0.000	0.017	0.004	0.020	0.000	0.000	0.000	0.000	0.000	0.004	0.000	0.004	0.001	0.004	0.004	0.000	0.004	0.001	0.005
Peru [5]	0.000	0.000	0.000	0.055	0.055	0.000	0.000	0.000	0.034	0.034	0.000	0.000	0.000	0.001	0.001	0.000	0.000	0.000	0.007	0.007	0.000	0.000	0.000	0.013	0.013
Suriname [5]	0.120	0.053	0.173	0.252	0.258	0.074	0.033	0.107	0.156	0.159	0.001	0.000	0.002	0.002	0.002	0.017	0.007	0.024	0.035	0.036	0.028	0.012	0.041	0.059	0.061
Venezuela [5]	0.000	0.002	0.002	0.001	0.002	0.000	0.001	0.001	0.001	0.001	0.000	0.000	0.000	0.000	0.000	0.000	0.000	0.000	0.000	0.000	0.000	0.000	0.000	0.000	0.000
French Guiana [5]	0.007	0.195	0.202	0.134	0.247	0.004	0.121	0.125	0.083	0.152	0.000	0.002	0.002	0.001	0.002	0.001	0.027	0.028	0.019	0.034	0.002	0.046	0.047	0.031	0.058
**SEARO**	16.638	20.946	37.574	47.974	48.174	11.019	14.008	25.020	31.879	32.005	0.306	0.401	0.707	0.911	0.914	2.208	2.776	4.983	6.348	6.375	3.105	3.760	6.863	8.836	8.881
Bangladesh [2]	0.463	1.469	1.932	4.716	4.716	0.316	1.003	1.319	3.221	3.221	0.012	0.039	0.052	0.127	0.127	0.058	0.185	0.244	0.595	0.595	0.076	0.241	0.317	0.773	0.773
Bhutan [2]	0.016	0.011	0.016	0.016	0.016	0.011	0.008	0.011	0.011	0.011	0.000	0.000	0.000	0.000	0.000	0.002	0.001	0.002	0.002	0.002	0.003	0.002	0.003	0.003	0.003
Burma [2]	1.230	0.055	1.285	1.362	1.400	0.726	0.032	0.758	0.804	0.826	0.016	0.001	0.017	0.018	0.018	0.162	0.007	0.169	0.179	0.184	0.327	0.015	0.341	0.362	0.372
India [2]	12.287	15.876	28.163	32.874	32.874	8.393	10.844	19.237	22.455	22.455	0.255	0.330	0.585	0.683	0.683	1.625	2.099	3.724	4.347	4.347	2.014	2.603	4.617	5.389	5.389
Indonesia [2]	2.005	2.395	4.399	6.303	6.360	1.183	1.413	2.596	3.719	3.752	0.015	0.018	0.032	0.046	0.047	0.275	0.329	0.604	0.865	0.873	0.532	0.636	1.168	1.673	1.689
Republic of Korea [2]	0.000	0.000	0.000	0.205	0.205	0.000	0.000	0.000	0.116	0.116	0.000	0.000	0.000	0.002	0.002	0.000	0.000	0.000	0.027	0.027	0.000	0.000	0.000	0.059	0.059
Nepal [2]	0.119	0.215	0.333	0.629	0.661	0.081	0.147	0.228	0.429	0.451	0.005	0.008	0.013	0.025	0.026	0.013	0.024	0.038	0.071	0.075	0.019	0.035	0.055	0.103	0.108
Sri Lanka [2]	0.038	0.162	0.200	0.225	0.277	0.026	0.111	0.136	0.154	0.189	0.000	0.001	0.001	0.001	0.002	0.006	0.024	0.029	0.033	0.040	0.006	0.027	0.033	0.037	0.045
Thailand [2]	0.414	0.764	1.178	1.600	1.600	0.244	0.451	0.695	0.944	0.944	0.002	0.004	0.007	0.009	0.009	0.058	0.106	0.164	0.222	0.222	0.110	0.203	0.313	0.425	0.425
Timor-Leste [2]	0.067	0.000	0.067	0.045	0.067	0.040	0.000	0.040	0.026	0.040	0.001	0.000	0.001	0.000	0.001	0.009	0.000	0.009	0.006	0.009	0.018	0.000	0.018	0.012	0.018

Table S2: Total number of pregnancies by pregnancy outcome in areas with P. falciparum and/or P. vivax transmission by continent in 2007 (in millions)

	Number of pregnancies at risk of malaria*†					Number of live-births born to pregnancies at risk of malaria* †					Number of stillbirths born to pregnancies at risk of malaria* †					Number of miscarriages among pregnancies at risk of malaria* †					Number of induced abortions among pregnancies at risk of malaria* †				
**2007**	*P. falciparum#*			*P. vivax#*	Any species	*P. falciparum#*			*P. vivax#*	Any species	*P. falciparum#*			*P. vivax#*	Any species	*P. falciparum#*			*P. vivax#*	Any species	*P. falciparum#*			*P. vivax#*	Any species
**Countries**	Stable¶	Unstable¶	Overall	Overall	Overall	Stable¶	Unstable¶	Overall	Overall	Overall	Stable¶	Unstable¶	Overall	Overall	Overall	Stable¶	Unstable¶	Overall	Overall	Overall	Stable¶	Unstable¶	Overall	Overall	Overall
**WPRO**	3.109	3.515	6.625	28.028	29.271	1.845	2.063	3.908	16.084	16.810	0.029	0.027	0.056	0.348	0.360	0.422	0.480	0.902	3.663	3.830	0.814	0.945	1.759	7.934	8.272
Cambodia [2]	0.445	0.105	0.550	0.522	0.596	0.262	0.062	0.325	0.308	0.352	0.005	0.001	0.007	0.006	0.007	0.059	0.014	0.073	0.069	0.079	0.118	0.028	0.146	0.139	0.158
China [2]	0.406	0.481	0.887	21.246	21.698	0.230	0.273	0.504	12.065	12.321	0.006	0.007	0.012	0.297	0.303	0.052	0.062	0.114	2.732	2.790	0.118	0.139	0.257	6.153	6.284
Lao [2]	0.214	0.001	0.215	0.094	0.215	0.126	0.000	0.127	0.055	0.127	0.003	0.000	0.003	0.001	0.003	0.028	0.000	0.028	0.012	0.028	0.057	0.000	0.057	0.025	0.057
Malaysia [2]	0.211	0.541	0.752	0.340	0.758	0.124	0.319	0.443	0.201	0.447	0.001	0.003	0.004	0.002	0.004	0.029	0.076	0.105	0.048	0.106	0.056	0.144	0.200	0.090	0.201
Papua New Guinea [3]	0.161	0.000	0.161	0.153	0.175	0.112	0.000	0.112	0.106	0.121	0.002	0.000	0.002	0.002	0.002	0.023	0.000	0.023	0.022	0.025	0.025	0.000	0.025	0.023	0.027
Philippines [2]	1.079	0.817	1.896	3.146	3.284	0.637	0.482	1.119	1.856	1.938	0.008	0.006	0.014	0.023	0.024	0.148	0.112	0.261	0.432	0.451	0.286	0.217	0.503	0.835	0.872
Solomon Islands [3]	0.017	0.000	0.017	0.015	0.019	0.012	0.000	0.012	0.010	0.013	0.000	0.000	0.000	0.000	0.000	0.002	0.000	0.002	0.002	0.003	0.003	0.000	0.003	0.002	0.003
Vanuatu [3]	0.008	0.000	0.008	0.008	0.009	0.006	0.000	0.006	0.005	0.006	0.000	0.000	0.000	0.000	0.000	0.001	0.000	0.001	0.001	0.001	0.001	0.000	0.001	0.001	0.001
Viet Nam [2]	0.568	1.570	2.138	2.505	2.517	0.335	0.926	1.261	1.478	1.485	0.004	0.011	0.015	0.017	0.017	0.078	0.216	0.294	0.345	0.347	0.151	0.417	0.568	0.665	0.668
**TOTAL**	**54.737**	**30.607**	**85.275**	**92.902**	**125.239**	**38.034**	**20.204**	**58.188**	**59.474**	**82.633**	**1.181**	**0.577**	**1.756**	**1.632**	**2.383**	**7.253**	**4.042**	**11.286**	**12.220**	**16.514**	**8.270**	**5.784**	**14.045**	**19.576**	**23.708**

* In millions

† The total number of pre

¥ TPR is the total numbe

Includes countries whe

¶ Stable transmission: >=

Abbreviation: TFR: Total

Note: The regional and t region and globally.

Continents: The numbe

* In millions

† The total number of pregnancies is the sum of the number of live-births, stillbirths, miscarriages and induced abortions.

Includes countries where P. falciparum and P. vivax co-exist

¶ Stable transmission: >= 0.1 autochthonous P. falciparum cases per 1,000 people per annum; unstable transmission <0.1 autochthonous P. falciparum cases per 1,000 people per annum

Note: The regional and total estimates for TFR, Stillbirth rate, TPR and pregnancy rates are weighted means. The regional and total numbers at risk was derived directly as the sum of the national estimates within each region and globally.

Continents: The number in superscript after each country's name refers to the continent to which the country belongs: 1 = Africa; 2=Asia; 3=Oceania; 4=North America; 5=South America; 6=Europe
