# Supplementary material for: Quantifying the Number of Pregnancies at Risk of Malaria in 2007: A Demographic Study
Source: PLoS Med. 2010 Jan 26;7(1):e1000221. doi: 10.1371/journal.pmed.1000221 (PMC2811150; doi:10.1371/journal.pmed.1000221)
Supplement: Table S3 — Total population, number of pregnancies, and number of live-births born to pregnancies in malaria endemic countries by continent in 2007 (in millions). (0.28 MB PDF) [file pmed.1000221.s004.pdf]

Table S3: Total population, number of pregnancies and number of live-births born to pregnancies in malaria endemic countries by continent in 2007 (in millions)

Continents	**Demographic data** Malaria Endemic Countries										**Total population at risk** (% of the population in Malaria Endemic Countries at risk)				
											P. falciparum transmission#			*P. vivax* transmission#	Any species
	Number of Malaria Endemic Countries	Total Population (both sexes)*	WOCBAs *	Total number of Pregnancie¥	TPR §	Pregnancy rate per 1000 WOCBAs §	Percentage pregnancies ending in:				Stable transmission¶	Unstable transmission ¶	Overall	Overall	Overall
							Live-births	Still-births	Spont-aneous Abortions	Induced Abortions					
Africa[1]	46	803	190	38.5	7.12	198	72.1%	2.4%	13.3%	12.2%	637.6 (79.4)	16.2 (2.0)	652.8 (81.2)	85.6 (10.7)	660.8 (82.2)
Asia[2]	28	3,726	984	105.8	3.76	135	62.5%	1.6%	13.1%	22.8%	702.2 (18.8)	918.7 (24.7)	1620.5 (43.5)	2975.3 (79.9)	3,048.2 (81.8)
Oceania[3]	3	7	2	0.3	5.46	156	69.3%	1.2%	14.2%	15.3%	4.8 (67.5)	0.0 (0.0)	4.8 (67.5)	4.5 (63.4)	5.2 (73.3)
N. America[4]	10	167	45	4.9	3.84	127	66.5%	0.8%	14.3%	18.4%	14.4 (8.6)	13.0 (7.8)	27.4 (16.4)	30.1 (18.1)	50.6 (30.3)
S. America[5]	11	363	98	10.6	3.79	122	61.8%	0.8%	13.9%	23.5%	26.8 (7.4)	37.2 (10.2)	64.0 (17.6)	66.1 (18.2)	87.6 (24.1)
Europe[6]	4	91	25	2.1	2.95	70	69.3%	0.9%	14.5%	15.3%	0.0 (0.0)	0.0 (0.0)	0.0 (0.0)	15.3 (16.8)	15.3 (16.8)
**Global**	**102**	**5,157**	**1,343**	**162.3**	**4.23**	**159**	**65.5%**	**1.8%**	**13.3%**	19.5%	**1385.8 (26.9)**	**985.1 (19.1)**	**2369.4 (45.9)**	**3176.9 (61.6)**	**3,867.6 (75.0)**

Continents	**Number of pregnancies at risk of malaria (column %)**					**Number of live-births born to pregnancies at risk of malaria (column %)**				
	P. falciparum transmission#			*P. vivax* transmission#	Any species	*P. falciparum* transmission#			*P. vivax* transmission#	Any species
	Stable transmission¶	Unstable transmission¶	Overall	Overall	Overall	Stable transmission¶	Unstable transmission¶	Overall	Overall	Overall
Africa[1]	31.3 (57.3)	0.7 (2.3)	32.0 (37.5)	4.3 (4.6)	32.4 (25.8)	22.8 (59.8)	0.5 (2.4)	23.2 (39.9)	3.0 (5.0)	23.5 (28.4)
Asia[2]	21.9 (39.9)	28.3 (92.5)	50.2 (58.8)	85.2 (91.7)	88.0 (70.3)	14.3 (37.6)	18.7 (92.6)	33.0 (56.7)	54.3 (91.2)	56.0 (67.8)
Oceania[3]	0.2 (0.3)	0.0 (0.0)	0.2 (0.2)	0.2 (0.2)	0.2 (0.2)	0.1 (0.3)	0.0 (0.0)	0.1 (0.2)	0.1 (0.2)	0.1 (0.2)
N. America[4]	0.6 (1.0)	0.5 (1.6)	1.1 (1.2)	0.9 (1.0)	1.7 (1.4)	0.4 (0.9)	0.3 (1.6)	0.7 (1.2)	0.6 (1.0)	1.1 (1.4)
S. America[5]	0.8 (1.4)	1.1 (3.6)	1.9 (2.2)	2.0 (2.1)	2.6 (2.1)	0.5 (1.3)	0.7 (3.4)	1.2 (2.0)	1.2 (2.1)	1.6 (1.9)
Europe[6]	0.0 (0.0)	0.0 (0.0)	0.0 (0.0)	0.4 (0.4)	0.4 (0.3)	0.0 (0.0)	0.0 (0.0)	0.0 (0.0)	0.2 (0.4)	0.2 (0.3)
**Global**	**54.7**	**30.6**	**85.3**	**92.9**	**125.2**	**38.0**	**20.2**	**58.2**	**59.5**	**82.6**

* Source: United Nations Development Program
¥ The total number of pregnancies is the sum of the number of live-births, stillbirths, spontaneous and induced abortions.
§ The total pregnancy rate (TPR) and the annual pregnancy rate per 1000 WOCBAs are weighted means per region and is for illustration purposes only. The number of pregnancies at risk was derived directly as the sum of the national estimates within each region and globally. They differ slightly from similar estimates obtained indirectly by use of the weighted regional or global estimates for pregnancy rates.
# Includes countries where *P. falciparum* and *P. vivax* co-exist
¶ Stable transmission: >= 1 autochthonous *P. falciparum* cases per 10,000 people per annum; Unstable transmission <1 autochthonous *P. falciparum* cases per 10,000 people per annum [14]
Abbreviation: N. America: North America; S. America: South America; MEC: Malaria Endemic Countries; TPR: Total Pregnancy Rate; WOCBA: Women of Childbearing Age (15-49 years of age).
Continent information: see table S1 for the countries included under the different continent and WHORO where Africa is denoted by 1; Asia by 2; Oceania by3; North America 4 , South America 5 and Europe 6
Regions information: The Africa and Europe regions are defined as the respective continents; Americas is defined as ‘North America’ plus 'South America' countries and Asia region defined as Asia-Pacific and Oceania combined
